# Typologies of suicidality and suicide presenting to a prehospital South African Emergency Medical Service: a retrospective cross-sectional analysis

**DOI:** 10.12688/f1000research.171712.1

**Published:** 2025-11-03

**Authors:** Daniel Tilley, Lloyd Denzil Christopher, Thomas Farrar, Navindhra Naidoo

**Affiliations:** 1 Emergency Medical Science, Cape Peninsula University of Technology Faculty of Health and Wellness Sciences, Cape Town, Western Cape, South Africa; 2 Mathematics and Physics, Cape Peninsula University of Technology Faculty of Applied Sciences, Cape Town, Western Cape, South Africa; 3 Paramedicine, School of health sciences, Western Sydney University Humanitarian and Development Research Initiative, Penrith, New South Wales, Australia

**Keywords:** Suicide and Suicidality, Typology, Syndemic research, Emergency Medical Service, Paramedicine

## Abstract

**Background:**

The global age-standardised suicide rate is estimated at 8.9/100 000, while South Africa is at 23.5/100 000. The prehospital Emergency Medical Services is located within this burden of health. Emergency Care providers have a duty to assess, treat and transport healthcare consumers with suicidality, when attending to the suicide caseload. The aim was to appraise suicidality case frequency and typology and estimate the scope of the challenge faced by a jurisdictional emergency medical service and its care providers.

**Methods:**

Using a retrospective cross-sectional design and a novel data collection instrument, a census of three years of Ambulance Incident Management Records was undertaken in a rural district of the Western Cape, South Africa.

**Results:**

Of 413,712 records, 2,976 (N) mental health-related incidents were sampled. Fourteen percent (n = 412) were assessed to have descriptors of suicidal ideation (n = 227), attempted suicide (n = 83) or death by suicide (n = 102). There were, on average, 2.8 deaths by suicide per month over the 3-year study period in the Garden Route District. Women were reported to mostly ingest poison and overdose on medication, while men used strangulation and were 5 times more likely to die by suicide than women.

**Conclusion:**

This study estimates the prehospital suicide and suicidality burden for the Western Cape Government Emergency Medical Services, elucidating an under-researched health concern within the South African prehospital space. Further study is required on the risk of emergency care provider stigmatisation towards suicide and suicidality cases, while auditing the need to assess policy, praxis, medical surveillance, EC provider clinical capacity and victim needs and experiences. Suicidality and suicide in Southern Africa could require a ‘Syndemic’ approach for the emergency service to interrupt suicidality and advance professional relevance.

## Highlights

• There were, on average, 2.3 and 2.8 attempted suicides and deaths by suicide in the Garden Route District over the 3-year study period, respectively ( Tilley et al., 2023).
• Women were found to use poison or medication overdose in suicide, while men used strangulation, presenting 5 times more likely to succumb to suicide than women ( Tilley et al., 2023).
• Future research is needed into suicidality and suicide management, praxis, policy and stigmatisation of suicidality victims from a prehospital EMS perspective. Exploring syndemic research on suicidality and its intersection with emergency care is warranted.

## Introduction

Death by Suicide in South Africa (SA) accounted for more insurance death claims than trauma, crime and motor vehicle accidents in 2024 ( Bhana, 2025). Globally, suicide accounted for 727 000 deaths in 2021-with an estimated 20 attempted suicides per suicide ( Caulkins, 2022)-more than war, homicide and HIV/AIDS, equating to one suicide every 100 deaths ( World Health Organization, 2025). The global age-standardised suicide rate in 2021 was estimated at 8.9 per 100 000, whereas the Africa region recorded 11.5 per 100 000. SA recorded 23.5 per 100 000 population, equating to almost 14 000 deaths by suicide per annum, placing SA third highest in suicide rates in Africa ( World Health Organization, 2024, 2025). Significantly, suicide was the third leading cause of death amongst people aged 15-29, while globally, suicide ranked as the 21 ^st^ leading cause of death in 2021 ( World Health Organization, 2024, 2025). Suicide and suicidality have become a public health burden and an under-researched priority in low-and middle-income countries ( Pompili, 2022).

SA endures a quadruple disease burden, which is antagonised by poverty, crime and inequality ( Ataguba et al., 2015; Burns, 2011) while producing numerous societal-level socioeconomic risk factors for mental illness exacerbation ( Motsoaledi & Matsoso, 2013). SA is deeply embedded in the social determinants of health which indirectly affects the social determinants of mental health ( Compton & Shim, 2017) with 63% of South Africans living in poverty; 31.9% unemployed; a rape case estimated at every 12minutes; 86 murders, 88 attempted murders and 595 assault cases daily and 18.9% of the population abusing alcohol and drugs ( Action Society, 2024; Egenasi et al., 2024; Gass et al., 2010; Myers et al., 2022; Prinsloo et al., 2022; The World Bank, 2024; World Bank Group, 2020). These conditions are all social antecedents for suicidality and suicide. Deinstitutionalisation through the Mental Health Care Act 17 of 2002 provided no compensatory mental health care, community service or prioritisation of mental health through a health care plan at a provincial level ( Burns, 2011; Lund et al., 2011, 2012), relying on the prehospital Emergency Medical Services (EMS) as a ‘primary’ health care point for all poor mental health sequalae and related emergencies ( Tilley, 2021; Van Huyssteen, 2016). This suggests that all South African prehospital Emergency Care (EC) Providers are required to assess, treat and transport mental health care consumers with suicidality by navigating their social determinants of mental health while experiencing a mental health treatment gap. The lack of mental health care consumer compliance is compounded by the loss of trained EC providers, psychologists, psychiatrists and mental health care providers ( Bateman, 2015; Burns, 2011; Jacob & Coetzee, 2018; Majiet et al., 2025).

These nuanced social constructs aid in the exacerbation of poor mental health, expediting pathways to suicidality. Notably, SA has multi-cultural challenges which can aggravate the need for urgent western mental health care ( Kirmayer, 2022; Kootbodien et al., 2020; Maharajh & Abdool, 2005; Motsoaledi & Matsoso, 2013), and complicate health seeking behaviour further. Caulkins (2022) posits a new premise whereby suicide is looked at as ‘ *syndemic’*, rather than syndromic, and illuminates intersectionality as an interdisciplinary technique to advance further understanding of suicidality behaviours ( Caulkins, 2022). Syndemic theory elucidates how combining cultural factors and two or more physiological factors manifests a public health challenge and builds on the social determinants of health theory ( Caulkins, 2022). Notwithstanding that research on suicide and suicidality has low reporting rates in Africa ( World Health Organization, 2025), most research is not done in the prehospital setting. The question that arises is: What is the typology of suicidality within the prehospital, rural EMS context? By appraising suicidality typology and epidemiologically descriptive evidence, the scope of the concern for the prehospital space becomes apparent. This has the potential to influence the building of suicidality capacity and knowledge for EC provider praxis.

## Materials and methods

### Design

Focusing on health care consumers with mental health needs was the pivot for this study. Using a retrospective cross-sectional design, data were extracted from the Western Cape Government Emergency Medical Services (WCGEMS) health care consumers’ Incident Management Records (IMR) from the Garden Route District, Western Cape (South Africa) from 2017 to 2019 (3 years). A census (100% sample) was taken of IMRs in the WCGEMS database that met the study’s inclusion criteria based on incident type. The dataset thus consisted of archival data related to health care consumers needing ambulance transport to a psychiatric facility, having psychiatric problems, overdosed, self-harmed or died by suicide. These incident types are routinely described by dispatchers/staff as ‘Self-Harm-other’, ‘Self-Harm-poisoning’, ‘Psychiatric/Behavioural Problems’ and ‘Inter-facility transfer (IFT)-psychiatric/behavioural problem’ ( Tilley et al., 2023). A census of these incident types included all EMS IMR from 2017 to 2019. IMRs associated with accidental poisoning of children under 8 years were excluded. IMRs are created by emergency call-takers and emergency ambulance dispatchers for every health care consumer who requires the WCGEMS and is the property of the WCGEMS Emergency Communications Centre ( Tilley, 2021; Tilley et al., 2023).

### Study setting and population

The study site was the rural Garden Route District, one of six district municipalities in the Western Cape. The Garden Route District comprises of seven local municipalities that experience poor socioeconomic conditions ( Tilley et al., 2023; Western Cape Government, 2019). The census approach identified a total of 413 712 IMRs from health care consumer interactions between 2017 and 2019; of these, 2 976 (N) met the incident type inclusion criteria. Of the 2 976 IMRs included in the dataset, 412 (n) health care consumers presented with suicidal ideation, attempted suicide and death by suicide. We provide this sub-group analysis here.

### Data analysis

Binary and multinomial logistic regression, Pearson’s Chi-squared test of independence, Fisher’s Exact Test, Analysis of Variance (ANOVA), and Tukey’s Honest Significant Difference (HSD) method were used to illuminate associations of interest among attempted suicide and suicide victims. Data was analysed in R statistical software ( R Core Team, 2025). Logistic regression allowed for analysis of relationships between a categorical dependent variable and one or more independent (predictor) variables, which could be categorical (Gender) or numerical (Age). If the dependent variable is binary (e.g., Suicide or No Suicide), the model predicts the probability of the binary outcome using a log-odds link function ( Sperandei, 2014). A multinomial logistic regression model can be used if the dependent variable has more than two categories (e.g., method of suicide or attempted suicide). The model coefficient(s) p-value of a significance test indicates probable relationships between dependent and predictor variables, usually expressed as an expected odds ratio. Pearson’s Chi-square test of independence tests whether two categorical variables have any association ( Bolboacă et al., 2011; Tilley, 2021). The null hypothesis (H _0_) states ‘there is no association between two variables while the alternative hypothesis (H _a_) states, there is an association between two variables ( Bolboacă et al., 2011, p. 530; Tilley, 2021; Tilley et al., 2023). Fisher’s Exact Test is another method for testing for an association between categorical variables, but unlike Pearson’s test, it does not rely on an asymptotic null distribution and thus the required assumptions are weaker ( Bolboacă et al., 2011; Nowacki, 2017). In the case of binary variables, one can use Fisher’s Exact Test to test for a directional alternative (i.e., a positive or negative association); ( Freeman & Campbell, 2007; Nowacki, 2017). For all hypothesis tests, we used a significance level of 0.05, meaning that if the *p*-value was less than 0.05, we rejected the null hypothesis; otherwise, the null hypothesis was retained ( Bolboacă et al., 2011; Nowacki, 2017).

A multinominal logistic regression model, ANOVA and Tukey’s HSD method were used to find smaller associations between gender, age and method of attempted suicide or suicide. ANOVA is used to show differences between two or more components through significance tests, making comparisons between populations ( Hosmer & Lemeshow, 2000; Sawyer, 2009; Tilley, 2021). The ANOVA test compares variation between sample means and variation within each of the samples. Low p-values are indications of compelling evidence against the null hypothesis that the group means are all equal. Tukey’s HSD method is based on a studentized range statistic and is used in connection with ANOVA as a post hoc method to identify pairwise significant differences, since the ANOVA test is an omnibus test that only identifies the presence or absence of differences in mean between treatments ( Hilton, 2006).

### Consent

A waiver of informed consent for a retrospective study was granted by an ethics committee, duly registered by the Western Cape Government National Department of Health Ethics Council *(WC_201911_033)*, as it was not practicable to obtain individual consent. There are adequate safeguards for participant privacy as all retrospective data were de-identified and there were no human participants engaged with during the data analysis. Consent was attained from the Western Cape Government Health and Wellness Emergency Medical Services (Ambulance Service) as custodian of the data.

## Results

Over the 3-year period of sampled health care consumers who presented to the WCGEMS, 14% (n = 412) presented with suicidality and death by suicide, while 63% (n = 1890) presented with mental illness sequela, considered stereotypical mental illness antecedents associated with suicide victims ( Kułak-Bejda et al., 2021). These were *overdose/DSP, substance abuse, depression, anxiety, self-harm, bipolar disorder, schizophrenia* and *PTSD* ( Table 1). There were, on average 2.8 deaths by suicide (n = 102) and 2.3 attempted suicides (n = 83) per month in the Garden Route District between 2017 and 2019. Gender and age associations were used to illuminate the suicidality and the death by suicide case load burden that EC providers from the WCGEMS face. Prehospital EC providers were expected to respond to 412 (n) suicidality and death by suicide health care consumers from the census population of 2 976 (N) emergencies over the 3-year period ( Tilley, 2025).

**Table 1.** Emergency Medical Service (EMS) Mental Illness typology.

Category	Frequency (n)	Relative frequency (%)
Overdose/DSP	1550	52%
Suicidal Ideation	227	7.6%
Substance Abuse	108	3.6%
Suicide	102	3.4%
Depression	89	3%
Attempted Suicide	83	2.7%
Anxiety	59	2%
Cutting Self-Harm	41	1.3%
Bipolar Disorder	21	0.7%
Schizophrenia	19	0.6%
Post-traumatic stress disorder	3	0.1%

Table 1 presents the counts of health care consumers with mental illness sequela that presented to the Western Cape Government Emergency Medical Services (WCGEMS) over a 3-year period (2017-2019). Using R statistical software, analysis found that Fourteen percent (n = 412) presented with suicidality and death by suicide, while sixty three percent (n = 1890) presented with mental illness sequela from the census population of 2 976 (N) emergencies over the 3-year period. There were, on average 2.8 deaths by suicide (n = 102) and 2.3 attempted suicides (n = 83) per month in the Garden Route District between 2017 and 2019. This typology consisted of *Overdose/Deliberate Self-Poison (DSP); Suicidal Ideation; Substance Abuse; Suicide; Depression; Attempted Suicide; Anxiety; Cutting Self-Harm; Bipolar Disorder; Schizophrenia* and *Post-traumatic stress disorder (PTSD).*

### ‘Death by Suicide Typology’

‘Death by Suicide’ was detected in 102 (n) of 2976 (N), suggesting 34 deaths by suicide per year of the study period ( Tilley et al., 2023). Death by Suicide was defined by types of method, namely, *‘Strangulation death’, ‘Overdose/DSP’, ‘jump from height’, ‘Gunshot’* and *‘Cutting Self-Harm’* ( Table 2). These cases precede postmortem and are service categories of the prehospital Emergency Medical Service and not that of the forensic pathologist.

**Table 2.** Frequency of method per Death by Suicide and Attempted Suicide.

Method	Death by Suicide (n)	Attempted Suicide (n)
Strangulation	82	51
Overdose/DSP	11	11
Jump *	4	2
Cutting Self-Harm	1	5
Gunshot	1	0
Unspecified	0	8
Parasuicidal	0	6

Table 2 presents the counts of health care consumers from the 3-year study period (2017-2019) who died by suicide and attempted suicide. From 2 976 (N) emergencies in the census sample, 102 (n) health care consumers died by suicide and 83 (n) attempted suicide. The methods that were used were *Strangulation (asphyxiation); Overdose/Deliberate Self-Poison; Jump from heigh* and *jump from moving vehicle; Cutting Self-Harm; Gunshot; Unspecified method* and *Parasuicidal.*

Death by Suicide caseload precedes postmortem and are service categories of the prehospital Emergency Medical Services and not that of the forensic pathologist.

* Jump refers to “jump from height” for Death by Suicide victims and “jump from moving car” for Attempted Suicide victims.

### ‘Attempted Suicide Typology’

‘Attempted Suicide’ was detected in 83 (n) of 2976 (N), suggesting 27 attempted suicides per year of the study period ( Tilley et al., 2023). The types of methods that are defined as attempted suicide are *‘Strangulation death’, ‘Overdose/DSP’, ‘jump from moving vehicle’, ‘Cutting Self-Harm’, ‘Parasuicidal’* (attempted suicide with no intention of death) and *‘Unspecified’* ( Table 2).

### Age and gender typology with attempted suicide and death by suicide

**Figure 1.**
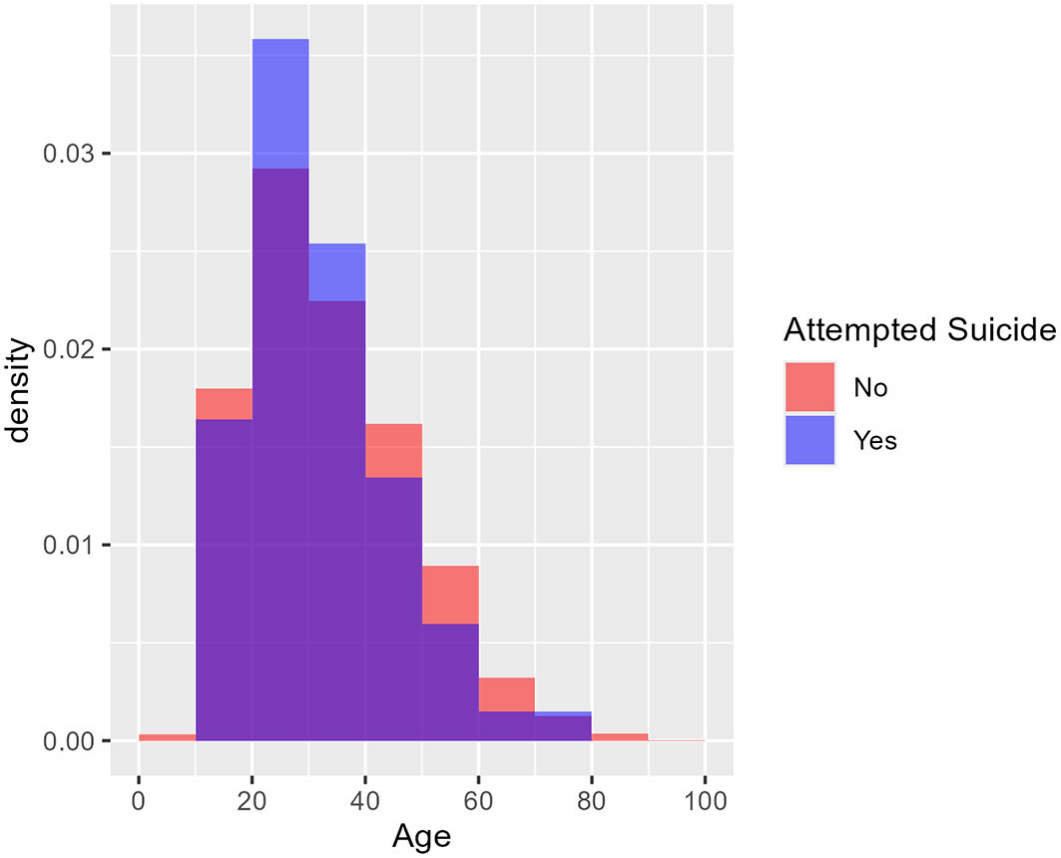
Age distribution of health care consumers who did and did not attempt suicide. Figure 1 shows the age distribution of health care consumers who had an had not attempted suicide using two overlaid histograms. From the logistic regression findings, the purple area in each plot denotes overlap between the two overlaid histograms while there is no visible difference between the red and blue histograms, suggesting no visible difference in age distribution between those who had and had not attempted suicide. Age was not a predictor of occurrence for ‘Attempted Suicide’ (p = 0.3095). From 3 years (2017-2019) of retrospective data, the median age for attempted suicide was 30 years old.

**Figure 2.**
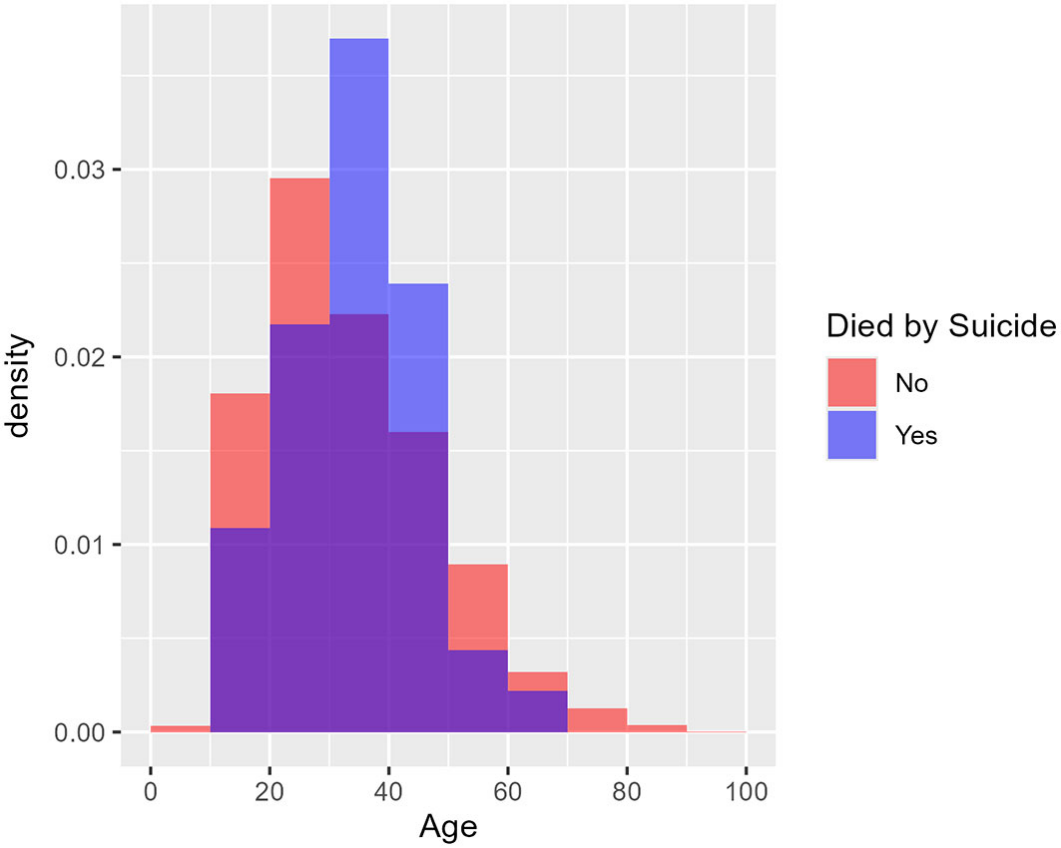
Age distribution of health care consumers who did and did not die by suicide. Figure 2 shows the age distribution of health care consumers who did and did not die by suicide using two overlaid histograms. From the logistic regression findings, purple area in each plot denotes overlap between the two overlaid histograms. Unlike Figure 1, the blue histogram’s density is concentrated in the middle, suggesting that health care consumers who died by suicide were particularly concentrated in the 30-50 age group. Age was not a predictor of occurrence for ‘Suicide’ ( *p = 0.3089*), however using a quadratic age term to check for non-monotonic relationships it was found in the ‘Suicide’ model, both the linear ( *p =* 0.00388) and quadratic ( *p =* 0.00681) terms were statistically significant. This suggests that the risk of death by suicide among health care consumers increases with age until a peak age of 41 and decreases thereafter. From 3 years (2017-2019) of retrospective data, the median age for suicide was 36 years old.

Using a significance level of *0.05* throughout the study, gender and age associations with ‘Death by Suicide’ and ‘Attempted Suicide’ provided further insight into this burden faced by prehospital EC providers working for the WCGEMS. Logistic regression, with age as the independent variable and ‘Attempted Suicide’ and ‘Death by Suicide’ as dependent variables, were run. Significantly, age was not a predictor of occurrence for ‘Death by Suicide’ ( *p = 0.3089*) or ‘Attempted Suicide’ ( *p = 0.3095*). However, the models were rerun with a quadratic age term to check for non-monotonic relationships. While there were still no significant effects in the ‘Attempted Suicide’ model ( *p* = 0.577 on the quadratic term), in the ‘Death by Suicide’ model, both the linear ( *p =* 0.00388) and quadratic ( *p =* 0.00681) terms were statistically significant. The fitted regression equation was $\log\frac{\pi }{1-\pi }=-8.241+0.2281x-0.00281x^{2}$ , where π is the probability of death by suicide and *x* is age. Using differential calculus, the function was maximised with respect to age, and it was thereby estimated that the age at which death by suicide risk is highest, is 41. This suggests that the risk of death by suicide among health care consumers increases with age until a peak age of 41 and decreases thereafter. The median ages for death by suicide and attempted suicide were 36 years and 30 years, respectively. Figure 1 shows the age distribution of health care consumers who had and had not attempted suicide using two overlaid histograms. Figure 2 similarly shows the age distribution of health care consumers who had and had not died by suicide. The purple area in each plot denotes overlap between the two overlaid histograms. The two figures cohere with the logistic regression findings: there is no visible difference between the red and blue histograms in Figure 1; hence no evidence of a difference in age distribution between those who attempted suicide and those who did not. In Figure 2, however, the blue histogram’s density is concentrated in the middle, suggesting that health care consumers who died by suicide were particularly concentrated in the 30-50 age group.

Using the Pearson Chi-square test of independence, it was found that the *p*-value was *< 0.05* for the associations between gender and attempted suicide ( *p = 0.004484*) and gender and death by suicide ( *p = 1.716 × 10 ^−8^*), suggesting males are more likely than females to die by suicide and attempt suicide. A logistic regression model was also fitted, with gender as the independent variable. Gender was again found to be a statistically significant predictor of both attempted suicide ( *p* = 0.00362) and death by suicide ( *p =* 1.78 × 10 ^−7^). The logistic regression model also allowed for computation of expected odds ratios. The odds of males attempting suicide were found to be 2.053 times as high as the odds of females attempting suicide, while the odds of males dying by suicide were found to be 5.049 times as high as those of females ( Tilley et al., 2023).

To analyse possible relationships between gender and age and the method of (attempted) suicide, cases of attempted suicide and death by suicide were combined to increase the frequencies. There were then 133 cases of strangulation, 22 cases of overdose or poisoning, and 28 cases of other or unspecified methods. Due to this response variable having three categories, a multinomial logistic regression model ( Table 3) was fitted with method of death by suicide/attempted suicide as a response variable and age and gender as independent variables, with an interaction of age and gender as well. No statistically significant coefficient predictors were found in the model at the 5% level. Looking at the method of death by suicide/attempted suicide vs. gender using Fisher’s Exact Test ( Table 4), there was a statistically significant relationship ( *p = 0.0005098*), specifically, it appears that males are more likely to use strangulation, while females are more likely to use poisoning or overdose.

**Table 3.** Multinomial logistic regression to predict death by suicide or attempted suicide method by age and gender.

*Coefficients:*				
**Method**	**(Intercept)**	**Age**	**Male**	Age * Male
*Other or unspecified*	-1.278676	0.02476155	0.9989150	0.01170249
*Strangulation death*	1.704831	-0.03863174	0.5557824	0.04890228
*Std. Errors:*				
**Method**	**(Intercept)**	**Age**	**Male**	Age * Male
*Other or unspecified*	1.2664027	0.02880904	2.380916	0.06678731
*Strangulation death*	0.9889576	0.02636292	2.006309	0.05978044
*p-values *				
**Method**	**(Intercept)**	**Age**	**Male**	Age * Male
*Other or unspecified*	0.3126430	0.3900612	0.6748137	0.8609066
*Strangulation death*	0.0847319	0.1428169	0.7817668	0.4133392

Table 3 presents the results from a multinominal logistic regression model used to predict death by suicide or attempted suicide method by age and gender. To analyse possible relationships between gender and age and the method of (attempted) suicide, cases of attempted suicide and death by suicide were combined to increase the frequencies. The multinomial logistic regression model was fitted with method of suicide/attempted suicide as a response variable and age and gender as independent variables, with an interaction of age and gender as well. No statistically significant coefficient predictors were found in the model at the 5% level. There were 133 cases of strangulation, 22 cases of overdose or poisoning and 28 cases of other or unspecified method.

**Table 4.** Fisher’s Exact Test – Gender association to type of Death by Suicide.

Gender	Overdose/poisoning	Other/Unspecified	Strangulation Death
Female	59.1%	35.7%	15.8%
Male	22.7%	35.7%	48.1%
Unknown	18.2%	28.6%	36.1%

p-value = 0.0005098; Alternative hypothesis: two.sided.

Table 4 presents the results of Fisher’s Exact Test, used to test for an association between gender and type of death by suicide, for the 3-year (2017-2019) census data. Looking at the method of death by suicide/attempted suicide vs. gender using Fisher's Exact Test, there was a statistically significant relationship ( *p = 0.0005098*), specifically, it appears that males are more likely to use strangulation, while females are more likely to use poisoning or overdose from the study site.

An analysis of variance (ANOVA) was also run to check for differences in mean age of victims (dependent variable) across the three methods of death by suicide or attempted suicide ( Table 5). Statistically significant differences between groups were identified ( *p = 0.0333*). Using Tukey’s HSD method for post hoc comparisons ( Table 6), it was found that the mean age of strangulation victims is less than the mean age of “Other or unspecified” victims ( *p = 0.0414684*) ( Tilley, 2025).

**Table 5.** ANOVA – Age relationship with method of Attempted Suicide or Death by Suicide.

Term	Degree of Freedom	Sum of Squared Residuals	Mean Squared	F-value	p-value
Method of Death by Suicide	2	1017.113	508.5565	3.509373	0.0333395
Residuals	109	15795.601	144.9138		

Table 5 presents results for an Analysis of Variance (ANOVA) that was run to check for differences in mean age of victims (dependent variable) across the three methods of death by suicide or attempted suicide. Statistically significant differences between groups were identified at *p = 0.0333* from the census data over the 3-year period (2017-2019).

**Table 6.** Tukey’s HSD Results - Differences in Mean Age by Method of Attempted Suicide or Death by Suicide.

Comparison	Difference in Means	Lower Confidence Limit	Upper Confidence Limit	Adjusted p-value
Other or unspecified-overdose or poisoning	2.750000	-6.543301	12.0433011	0.76215151
Strangulation death-overdose or poisoning	-4.689189	-12.206652	2.8282732	0.30343063
Strangulation death-other or unspecified	-7.439189	-14.647982	-0.2303966	0.04146837

Table 6 presents the results for Tukey’s Honest Significant Difference (HSD) test. This method is based on a studentized rage statistic and is used in connection with analysis of variance (ANOVA) as a post hoc method to identify pairwise significant differences. Using Tukey’s HSD method for post hoc comparisons, it was found that the mean age of strangulation victims is less than the mean age of “Other or unspecified” victims ( *p = 0.0414684*) from the census data over the 3-year period (2017-2019).

## Discussion

There was a range of death by suicide and suicidality typologies in the Garden Route District over the 3-year study period, presenting in 14% ( *n* = 412) of Incident Management Records sampled (representing health care consumer engagement). Death by suicide took place on average 2.8 ( *n *= 102) times a month, and attempted suicide 2.3 ( *n *= 83) times a month, and roughly 6.3 ( *n* = 227) health care consumers presented to the WCEMS with suicidal ideation monthly. Notably 63% ( *n* = 1890) of Incident Management Records (health care consumers) presented with mental illness sequela, often related to suicide and suicidality victims’ medical history ( Klonsky et al., 2016; Kułak-Bejda et al., 2021). Significantly, in the same sample, males were five and two times more likely to die by suicide and attempt suicide than females, respectively ( Tilley et al., 2023). Males appeared more likely to use strangulation, while females used overdose or poisoning as a means of death by suicide in the study site ( Tilley et al., 2023).

The data analysis provided similar inferences already denoted in articles on suicide globally, with strangulation (hanging) being the most common method, and men being the most likely to die by suicide ( Klonsky et al., 2016; Kootbodien et al., 2020; Rahman et al., 2017; Ritchie et al., 2015). However, the presence of suicidal ideation, attempted suicides, cutting self-harm and overdose/DSP in the dataset is what was illuminating. Understandably, antecedents for suicidality ( Klonsky et al., 2016; Lim et al., 2019), individuals who inflict non-suicidal self-injury (disorder) are at risk of suicide attempts ( Brager-Larsen et al., 2024), while suicidal ideation and progression into suicide attempts are two phenomena that produce predictors towards death by suicide ( World Health Organization, 2014). Using the ideation-to-action framework, literature suggests deliberate self-harm and depression to be early and accurate indicators for suicidal ideation and suicidality ( Klonsky et al., 2016). The mental illness sequela from the dataset suggests that, through better medical surveillance, more effort could be put into early suicide detection, knowing that PTSD, bipolar disorder, depression, substance abuse and suicidal ideation are associated with suicide deaths ( Klonsky et al., 2016). Prehospital emergency medical care provides health action to health conditions through emergency medicine in a time sensitive approach with universality and responsivity ( Christopher et al., 2014; Naidoo, 2017; Tilley et al., 2023) and can recognise the patterns and needs of mental health care consumers from an early stage to interrupt suicidality and limit access to methods of harm ( Florentine & Crane, 2010).

This dataset does not explain the perceptions prehospital EC providers have towards health care consumers who have suicidal ideation, suicidality and have attempted suicide. In various articles prehospital EC providers have explained to feel misconstrued on the concept of mental illness, self-harm and suicidality, often feeling lost and depleted by lack of legislation and policy, treatment protocols, training, guidance and personal negative conflict on own perceptions of attending mental health emergencies rather than trauma/medical emergencies ( Evans et al., 2018; O’Sullivan, 2014; Rees et al., 2015, 2018; Stander et al., 2021). In a study done in the same province in SA, it was found that 80% of the prehospital EC providers in the study had no prior training to manage suicidal health care consumers, seldom using formal suicide evaluation and capacity check tools, while implying negative feelings and connotations towards attempted suicide victims ( Evans et al., 2018). Inevitably, this lack of compassion, training and knowledge provides a precarious situation for prehospital EC providers, as this could prevent early mental health surveillance and suicidality interruption. A lack of praxis and management of suicidality and death by suicide caseload could put the EC provider helpless, creating an emotional backlash with vicarious traumatisation to the EC provider. Notably, prehospital EC providers have disclosed battling with lasting visions from death by suicide scenes and battling with anxiety, PTSD and depression ( Padmanabhanunni & Pretorius, 2025; Rothes et al., 2020).

WCGEMS prehospital EC providers locate in the suicide and suicidality burden and need to consider analytical clinical decision making ( Emond et al., 2024) in managing and treating mental health consumers while considering the societal, cultural, religious and socioeconomic risk factors synonymous with suicide in SA ( Kootbodien et al., 2020). SA is precariously placed in the mental health milieu, with deinstitutionalisation and no appropriate policy and compensatory community mental health services created ( Motsoaledi & Matsoso, 2013; World Health Organization, 2003). Atrocities from apartheid, poverty and inequality create exponential societal risk levels for suicidality and suicide, placing the prehospital EC provider and EMS at the forefront of mental health and suicidality emergencies. Located in the forefront of this caseload, prehospital EC providers need to have capacity to manage, treat and transport these health care consumers, have potential to interrupt suicidality by limiting access to harmful methods ( Florentine & Crane, 2010), contribute to social capital through latent capacity ( Tilley et al., 2023) while minding risk of direct and vicarious self-traumatization ( Sandford et al., 2021). The WCGEMS-embedded in the South African socioeconomic traumatic past should consider ‘Trauma Informed Care’ as an interlude to disrupt negative postulation to suicidality, whereby understanding that childhood traumatic experiences can show signs of future mental health challenges, as most (mental) health is affected by past trauma ( Melillo et al., 2025; SAMHSA, 2014).

## Conclusion

Tilley et al *.* (2023) described the Deliberate Self-Harm (DSH) burden for the prehospital EMS; however, the novelty in this paper provides and removes the DSH scope and focuses on prehospital suicidality typology while suggesting a syndemic approach to suicide in the African setting. The authenticity of this research elucidates the suicidality burden faced by the South African prehospital EMS. Death by suicide and suicidality typology in the EMS have not been previously assessed in South Africa, illuminating a problem space. This study describes the prehospital suicide and suicidality burden for the WCGEMS. Prehospital EC providers need to retain the praxis, training, emotion, policies and legislation to comprehensively manage, treat and transport health care consumers with suicidality ( Simpson et al., 2025) and that this proven challenge could require lateral deliberation ( Emond et al., 2024). It could be purposeful to consider ‘Trauma Informed Care’ ( SAMHSA, 2014) in an approach to management and training for health care consumers and EC providers. This could provide a dignified response to managing a marginalised group and the risk of vicarious traumatisation of the EC provider ( Sandford et al., 2021). This documented death by suicide and suicidality typology presents an understanding of the prehospital suicidality problem space definition. This study quantifies the burden for the EMS; however, it provides no solution to training, management, treatment or EC provider perception towards death by suicide and suicidality. Further study is required on EC provider stigmatisation towards death by suicide and suicidality, while auditing the need to assess policy, praxis, medical surveillance, EC provider clinical competency capacity and suicidality victim perspective, as health care consumer level interventions on strategic suicide prevention have aided in the reduction of suicide attempts ( Zarska et al., 2023). Suicidality and suicide in Southern Africa could require a ‘Syndemic’ approach ( Caulkins, 2022) for health authorities (such as EMS) to interrupt suicidality and be protective of people living with such risk.

### Limitations of the study

Emergency call takers are not trained mental health professionals and don’t make mental health diagnoses, while the vernacular of the health care consumers provides a challenge in reporting. Retrospective data limitations apply ( Tilley et al., 2023). This dataset does not elucidate perceptions of prehospital EC providers towards suicidality.

## Ethical considerations

Ethics was granted for this study through the ethical board of the Cape Peninsula University of Technology through a vigorous assessment of a research proposal *(CPUT/HW-REC 2019/H17).* Ethical permission for site approval was obtained from the Western Cape Government National Department of Health Ethics Council *(WC_201911_033),* granting approval to partake in quantitative retrospective research within the Western Cape Government Health and Wellness Emergency Medical Services. ( Tilley, 2021, 2025).

## Data Availability

Figshare: Access to health care for health care consumers with mental health needs: an Emergency Medical Service perspective. https://doi.org/10.6084/m9.figshare.30392569. The project contains raw retrospective data in an Excel spreadsheet with analysed data in graphs and tables using R statistical software, Binary and multinomial logistic regression, Pearson’s Chi-squared test of independence, Fisher’s Exact Test, Analysis of Variance (ANOVA), and Tukey’s Honest Significant Difference (HSD). A dissertation with full analysis is also provided. Data are available under the terms of the Creative Commons Attribution 4.0 International licence (CC-BY 4.0). ( Tilley, 2025)
