# Typologies of suicidality and suicide presenting to a prehospital South African Emergency Medical Service: a retrospective cross-sectional analysis

**DOI:** 10.12688/f1000research.171712.3

**Published:** 2026-03-04

**Authors:** Daniel Tilley, Lloyd Denzil Christopher, Thomas Farrar, Navindhra Naidoo

**Affiliations:** 1 Emergency Medical Science, Faculty of Health and Wellness Sciences,Cape Peninsula University of Technology, Cape Town, Western Cape, South Africa; 2 Mathematics and Physics, Faculty of Applied Sciences, Cape Peninsula University of Technology, Cape Town, Western Cape, South Africa; 3 Paramedicine, School of Health sciences//Humanitarian and Development Research Initiative (HADRI)/Young and Resilient Research Centre, Institute for Culture and Society,: Western Sydney University; Legitimation Code Theory Centre for Knowledge Building: University of Sydney, New South Wales, Australia

**Keywords:** Suicide and Suicidality, Typology, Syndemic research, Emergency Medical Service, Paramedicine

## Abstract

**Background:**

The global age-standardised suicide rate is estimated at 8.9/100 000, while South Africa is at an alarming 23.5/100 000. The prehospital Emergency Medical Services (EMS) is located within this burden of health need. Emergency Care providers have a duty to assess, treat and transport healthcare consumers with suicidality, when attending to the suicide-related caseload.

**Aim:**

To appraise suicidality case frequency and suicide typology for the EMS.

**Objectives:**

To estimate the scope of the suicidality challenge faced by a jurisdictional EMS and its care providers.

**Methods:**

Using a retrospective cross-sectional design and a novel data collection instrument, a census of three years of Ambulance Incident Management Records was undertaken in a rural district of the Western Cape, South Africa.

**Results:**

Of 413,712 records, 2,976 (N) mental health-related incidents were sampled. Fourteen percent (n = 412) were assessed to have descriptors of suicidal ideation (n = 227), attempted suicide (n = 83) or death by suicide (n = 102). There were, on average, 2.8 deaths by suicide per month over the 3-year study period in the Garden Route District. Women were reported to mostly ingest poison and overdose on medication, while men used asphyxiation/hanging and were 5 times more likely to die by suicide than women.

**Conclusion:**

This study estimates the prehospital suicide and suicidality burden for the Western Cape public Emergency Medical Services, elucidating an under-researched health concern within South African prehospital care. Further study is required on the risk of emergency care provider stigmatisation towards suicide and suicidality cases, while auditing the need to assess policy, praxis, medical surveillance, EC provider clinical capacity and victim needs and experiences.

## Highlights

• There were, on average, 2.3 and 2.8 attempted suicides and deaths by suicide in the Garden Route District over the 3-year study period, respectively.
• Women were found to use poison or medication overdose in suicide, while men used asphyxiation/hanging, presenting 5 times more likely to succumb to suicide than women.
• Future research is needed into suicidality and suicide management, praxis, policy and stigmatisation of suicidality victims from a EMS perspective. Exploring trauma informed care and a syndemic approach to suicidality intervention and intersections with prehospital emergency care may enhance system responses.

## Introduction

Death by suicide in South Africa (SA) accounted for more insurance-related death claims than trauma, crime and motor vehicle accidents in 2024 ( Bhana, 2025). Globally, suicide accounted for 727 000 deaths in 2021-with an estimated 20 attempted suicides per suicide ( Caulkins, 2022)-greater mortality than war, homicide and HIV/AIDS. This equates to one suicide for every 100 deaths ( World Health Organization, 2025). The global age-standardised suicide rate in 2021 was estimated at 8.9 per 100 000, whereas the Africa region recorded 11.5 per 100 000. SA recorded 23.5 per 100 000 population, equating to almost 14 000 deaths by suicide per annum, placing SA third highest in suicide rates in Africa ( World Health Organization, 2024, 2025). Significantly, suicide was the third leading cause of death amongst people aged 15-29, while globally, suicide ranked as the 21 ^st^ leading cause of death in 2021 ( World Health Organization, 2024, 2025). Suicide and suicidality have become a public health burden and an under-researched priority in low-and middle-income countries (LMICs) ( Pompili, 2022).

SA endures a quadruple disease burden, the effects of which are compounded by poverty, crime and inequality ( Ataguba et al., 2015; Burns, 2011) while producing numerous societal-level socioeconomic risk factors for mental illness exacerbation ( Motsoaledi & Matsoso, 2013). SA is deeply embedded in the social determinants of health, which indirectly affects the social determinants of mental health ( Compton & Shim, 2017). Sixty three percent of South Africans live in poverty; 31.9% are unemployed ( The World Bank, 2024; World Bank Group, 2020); a rape case is estimated to take place every 12 minutes ( Action Society, 2024; Egenasi et al., 2024; Gass et al., 2010); 86 murders, 88 attempted murders and 595 assault cases are reported to happen daily ( Prinsloo et al., 2022) and 18.9% of the population abuse alcohol and/or drugs ( Myers et al., 2022). These nuanced social constructs are all social antecedents for suicidality and suicide by aiding in the exacerbation of poor mental health, expediting pathways to suicidality.

Deinstitutionalisation through the Mental Health Care Act 17 of 2002 provided no compensatory mental health care community service or prioritisation of mental health through a health care plan at a provincial level ( Burns, 2011; Lund et al., 2011, 2012). Thus, EMS is *de facto* relied upon as a primary health care point for all poor mental health sequalae and related emergencies ( Tilley, 2021; Van Huyssteen, 2016). Notably, SA has multi-cultural challenges which can aggravate the need for urgent western mental health care ( Kirmayer, 2022; Kootbodien et al., 2020; Maharajh & Abdool, 2005; Motsoaledi & Matsoso, 2013), and complicate health seeking behaviour further. This suggests that all South African prehospital Emergency Care (EC) Providers are required to assess, treat and transport mental healthcare consumers with suicidality by navigating their social determinants of mental health while experiencing a mental health treatment gap. The lack of mental healthcare consumer compliance is further compounded by the loss of trained EC providers, psychologists, psychiatrists and mental healthcare providers ( Bateman, 2015; Burns, 2011; Jacob & Coetzee, 2018; Majiet et al., 2025).

Caulkins (2022) posits that suicide is looked at as ‘ *syndemic’*, rather than syndromic, and illuminates intersectionality as an interdisciplinary technique to advance further understanding of suicidality behaviours ( Caulkins, 2022). Syndemic theory elucidates how combining cultural factors and two or more physiological factors manifests a public health challenge and builds on the social determinants of health theory ( Caulkins, 2022). Considering syndemic theory as a theoretical lens for system level responses to suicide may have value for manifesting suicidality awareness from an African/LMIC perspective, notwithstanding that research on suicide and suicidality has low reporting rates in Africa ( World Health Organization, 2025) and is poorly represented in prehospital emergency care research.

There is limited empirical evidence describing suicidality typology in the South African prehospital EMS research. By appraising suicidality typology and epidemiologically descriptive evidence, the scope of the concern for the prehospital space becomes apparent. This has the potential to influence the building of suicidality capacity and knowledge for EC provider praxis. Thus, the question that arises is: What is the typology of suicidality within the prehospital, rural EMS context? The aim was to appraise suicidality case frequency and suicide typology for the EMS with the objectives being to estimate the scope of the suicidality challenge faced by a jurisdictional EMS and its care providers.

## Materials and methods

### Design

Focusing on healthcare consumers with mental health needs was the pivot for this study. Coherent with a critical theory paradigm ( Naidoo, 2011), a retrospective cross-sectional observational design, with quantitative analysis was used. Data were extracted from the Western Cape Department of Health and Wellness public Emergency Medical Services (WCEMS) healthcare consumers’ Incident Management Records (IMR) from the Garden Route District, Western Cape (South Africa) from 2017 to 2019 (3 years). A census (100% sample) was taken of IMRs in the WCEMS database that met the study’s inclusion criteria based on incident type. The dataset thus consisted of archival data related to healthcare consumers needing ambulance transport to a psychiatric facility, having psychiatric challenges, overdosed, self-harmed or died by suicide. ‘Died by suicide’ was defined as a prehospital service category and not as a forensic confirmation.

These incident types are recorded in the EMS database as ‘Self-Harm-other’, ‘Self-Harm-poisoning’, ‘Psychiatric/Behavioural Problems’ and ‘Inter-facility transfer (IFT)-psychiatric/behavioural problem’ ( Tilley et al., 2023). A census of these incident types included all EMS IMR from 2017 to 2019. IMRs associated with accidental poisoning of children under 8 years were excluded. IMRs are created by emergency call-takers and emergency ambulance dispatchers for every healthcare consumer who requires the WCEMS and is the property of the WCEMS Emergency Communications Centre ( Tilley, 2021; Tilley et al., 2023). Notably, emergency call-takers and emergency ambulance dispatchers are not mental health care professionals and rely on senior medical advice, caller descriptive prompts and confirmation of healthcare consumer triage from senior EC providers arriving on scene.

### Study setting and population

The study site was the rural Garden Route District, one of six district municipalities in the Western Cape. The Garden Route District comprises of seven local municipalities that experience poor socioeconomic conditions ( Tilley et al., 2023; Western Cape Government, 2019). The census approach identified a total of 413 712 IMRs from health care consumer interactions between 2017 and 2019; of these, 2 976 (N) met the incident type inclusion criteria. Of the 2 976 IMRs included in the dataset, 412 (n) healthcare consumers presented with suicidal ideation (thoughts of death), attempted suicide (having attempted death), and death by suicide (having died). Considering the vernacular, it is notable that an emergency caller would describe a healthcare consumer as ‘having thoughts of death’ (suicidal ideation), ‘having tried to kill oneself’ (attempting suicide) or ‘taken their own life’ (death by suicide) to emergency call takers when calling for help. We provide this sub-group analysis here.

### Data analysis

Binary and multinomial logistic regression, Pearson’s Chi-squared test of independence, Fisher’s Exact Test, Analysis of Variance (ANOVA), and Tukey’s Honest Significant Difference (HSD) method were used to illuminate associations of interest among attempted suicide and suicide victims. Data was analysed in R statistical software ( R Core Team, 2025). Logistic regression allowed for analysis of relationships between a categorical dependent variable and one or more independent (predictor) variables, which could be categorical (Gender) or numerical (Age). If the dependent variable is binary (e.g., Suicide or No Suicide), the model predicts the probability of the binary outcome using a log-odds link function ( Sperandei, 2014). A multinomial logistic regression model can be used if the dependent variable has more than two categories (e.g., method of [attempted] suicide). The model coefficient(s) p-value of a significance test indicates probable relationships between dependent and predictor variables, usually expressed as an expected odds ratio. Pearson’s Chi-square test of independence tests whether two categorical variables have any association ( Bolboacă et al., 2011). The null hypothesis (H _0_) states ‘there is no association between two variables while the alternative hypothesis (H _a_) states, there is an association between two variables ( Bolboacă et al., 2011, p. 530). Fisher’s Exact Test is another method for testing for an association between categorical variables, but unlike Pearson’s test, it does not rely on an asymptotic null distribution and thus the required assumptions are weaker ( Bolboacă et al., 2011; Nowacki, 2017). In the case of binary variables, one can use Fisher’s Exact Test to test for a directional alternative (i.e., a positive or negative association); ( Freeman & Campbell, 2007; Nowacki, 2017). For all hypothesis tests, we used a significance level of 0.05, meaning that if the *p*-value was less than 0.05, we rejected the null hypothesis; otherwise, the null hypothesis was retained ( Bolboacă et al., 2011; Nowacki, 2017).

A multinomial logistic regression model, ANOVA and Tukey’s HSD method were used to find smaller associations between gender, age and method of attempted suicide or suicide. ANOVA is used to show differences between two or more groups through significance tests, making comparisons between populations ( Hosmer & Lemeshow, 2000; Sawyer, 2009). The ANOVA test compares variation between sample means and variation within each of the samples. Low p-values are indications of compelling evidence against the null hypothesis that the group means are all equal. Tukey’s HSD method is based on a studentized range statistic and is used in connection with ANOVA as a post hoc method to identify pairwise significant differences, since the ANOVA test is an omnibus test that only identifies the presence or absence of differences in mean between treatments ( Hilton, 2006). Tukey’s method is designed to control the familywise type I error rate at a fixed level (e.g., 0.05) despite the large number of pairwise comparisons being made.

### Consent

A waiver of informed consent for a retrospective study was granted by an institutional ethics committee *(CPUT/HW-REC 2019/H17)*, duly registered by the National Health Research Ethics Committee, as it was not practicable to obtain individual consent. Access to the Western Cape National Health Research Database *(WC_201911_033)* was granted by the provincial health department. There are adequate safeguards for participant privacy as all retrospective data were de-identified and there were no human participants engaged with during the data analysis.

## Results

Over the 3-year period of sampled healthcare consumers who presented to the WCEMS, 14% (n = 412) presented with suicidality and death by suicide, while 63% (n = 1890) presented with mental illness sequela, considered stereotypical mental illness antecedents associated with deaths by suicide ( Kułak-Bejda et al., 2021). These were *overdose/DSP, substance abuse, depression, anxiety, self-harm, bipolar disorder, schizophrenia* and *PTSD* ( Table 1). There were, on average 2.8 deaths by suicide (n = 102) and 2.3 attempted suicides (n = 83) per month in the Garden Route District between 2017 and 2019. Gender and age associations were used to illuminate the suicidality and the death by suicide case load burden that EC providers from the WCEMS face. Prehospital EC providers were expected to respond to 412 (n) suicidality and death by suicide healthcare consumers from the census population of 2 976 (N) emergencies over the 3-year period ( Tilley, 2025).

**Table 1.** Emergency Medical Service (EMS) Mental Illness typology.

Category	Frequency (n)	Relative frequency (%)
Overdose/DSP	1550 (n)	52%
Suicidal Ideation	227 (n)	7.6%
Substance Abuse	108 (n)	3.6%
Suicide	102 (n)	3.4%
Depression	89 (n)	3%
Attempted Suicide	83 (n)	2.7%
Anxiety	59 (n)	2%
Cutting Self-Harm	41 (n)	1.3%
Bipolar Disorder	21 (n)	0.7%
Schizophrenia	19 (n)	0.6%
Post-traumatic stress disorder	3 (n)	0.1%

Table 1 presents the counts of health care consumers with mental illness sequela that presented to the Western Cape Government Emergency Medical Services (WCGEMS) over a 3-year period (2017-2019). Using R statistical software, analysis found that Fourteen percent (n = 412) presented with suicidality and death by suicide, while sixty three percent (n = 1890) presented with mental illness sequela from the census population of 2 976 (N) emergencies over the 3-year period. There were, on average 2.8 deaths by suicide (n = 102) and 2.3 attempted suicides (n = 83) per month in the Garden Route District between 2017 and 2019. This typology consisted of *Overdose/Deliberate Self-Poison (DSP); Suicidal Ideation; Substance Abuse; Suicide; Depression; Attempted Suicide; Anxiety; Cutting Self-Harm; Bipolar Disorder; Schizophrenia* and *Post-traumatic stress disorder (PTSD).*

### ‘Death by Suicide Typology’

‘Death by Suicide’ was detected in 102 (n) of 2976 (N), suggesting 34 deaths by suicide per year of the study period. Death by suicide was defined by types of method, namely, *‘Asphyxiation/hanging’, ‘Overdose/DSP’, ‘jump from height’, ‘Gunshot’* and *‘Cutting Self-Harm’* ( Table 2). These cases precede postmortem and are service categories of the prehospital EMS and not that of the forensic pathologist.

**Table 2.** Frequency of method per Death by Suicide and Attempted Suicide.

Method	Death by Suicide (n)	Attempted Suicide (n)
Asphyxiation/hanging	82 (n)	51 (n)
Overdose/DSP	11 (n)	11 (n)
Jump *	4 (n)	2 (n)
Cutting Self-Harm	1 (n)	5 (n)
Gunshot	1 (n)	0 (n)
Unspecified	0 (n)	8 (n)
Parasuicidal	0 (n)	6 (n)

Table 2 presents the counts of health care consumers from the 3-year study period (2017-2019) who died by suicide and attempted suicide. From 2 976 (N) emergencies in the census sample, 102 (n) health care consumers died by suicide and 83 (n) attempted suicide. The methods that were used were *Strangulation (asphyxiation); Overdose/Deliberate Self-Poison; Jump from heigh* and *jump from moving vehicle; Cutting Self-Harm; Gunshot; Unspecified method* and *Parasuicidal.*

Death by Suicide caseload precedes postmortem and are service categories of the prehospital Emergency Medical Services and not that of the forensic pathologist.

* Jump refers to “jump from height” for death by suicide victims and “jump from moving car” for attempted suicide victims.

### ‘Attempted Suicide Typology’

‘Attempted Suicide’ was detected in 83 (n) of 2976 (N), suggesting 27 attempted suicides per year of the study period ( Tilley et al., 2023). The types of methods that are defined as attempted suicide are *‘Asphyxiation/hanging’, ‘Overdose/DSP’, ‘jump from moving vehicle’, ‘Cutting Self-Harm’, ‘Parasuicidal’* (attempted suicide with no intention of death, dictated by repeated offences by the same healthcare consumer, flagged by consistent healthcare consumer pattern and labelled by authors) and *‘Unspecified’* ( Table 2).

### Age and gender typology with Attempted Suicide and Death by Suicide

Using a significance level of *0.05* throughout the study, gender, and age associations with ‘death by suicide’ and ‘attempted suicide’ provided further insight into this burden faced by prehospital EC providers working for the WCEMS. Logistic regression models, with age as the independent variable and ‘attempted suicide’ and ‘death by suicide’ as dependent variables, were run. Significantly, age was not a predictor of occurrence for ‘death by suicide’ ( *p = 0.3089*) or ‘attempted suicide’ ( *p = 0.3095*). However, the models were rerun with a quadratic age term to check for non-monotonic relationships. While there were still no significant effects in the ‘attempted suicide’ model ( *p* = 0.577 on the quadratic term), in the ‘death by suicide’ model, both the linear ( *p =* 0.00388) and quadratic ( *p =* 0.00681) terms were statistically significant. The fitted regression equation was logit(π) =−8.241+0.2281 x−0.00281 x ^2^, where π is the probability of death by suicide and *x* is age. Using differential calculus, the function was maximised with respect to age, and it was thereby estimated that the age at which death by suicide risk is highest, is 41. A similar logistic regression model was run where the dependent variable cases were classified as positive for attempted suicide *or* death by suicide. The linear ( *p =* 0.0299) and quadratic ( *p* = 0.0290) age terms were statistically significant in the resulting model, albeit with higher *p*-values than for the model that considered death by suicide only. The fitted model equation is logit(π) =−4.566+0.08383 x−0.001109 x ^2^. This model predicts that the age at which the risk of attempted suicide or death by suicide is maximized is 38, which is not very different from the result obtained from the model that considered death by suicide only.

**Figure 1.**
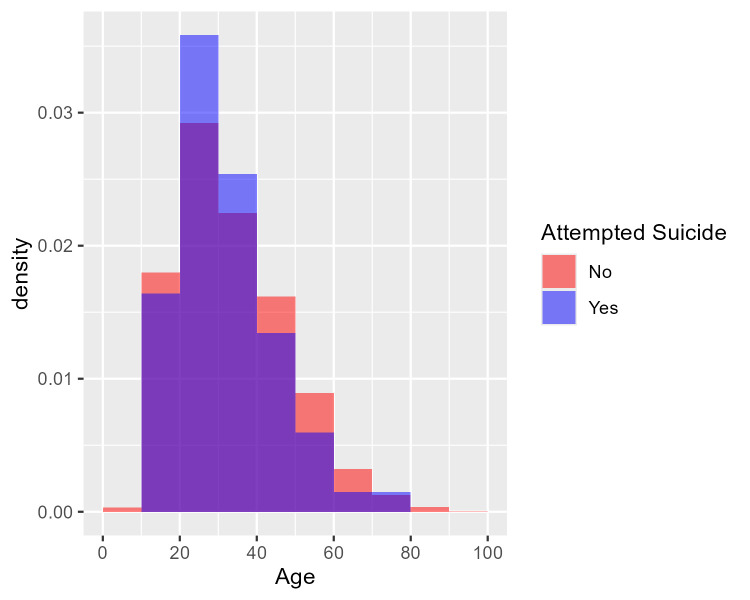
Age distribution of health care consumers who did and did not attempt suicide. Figure 1 shows the age distribution of health care consumers who had an had not attempted suicide using two overlaid histograms. From the logistic regression findings, the purple area in each plot denotes overlap between the two overlaid histograms while there is no visible difference between the red and blue histograms, suggesting no visible difference in age distribution between those who had and had not attempted suicide. Age was not a predictor of occurrence for ‘Attempted Suicide’ (p = 0.3095). From 3 years (2017-2019) of retrospective data, the median age for attempted suicide was 30 years old.

**Figure 2.**
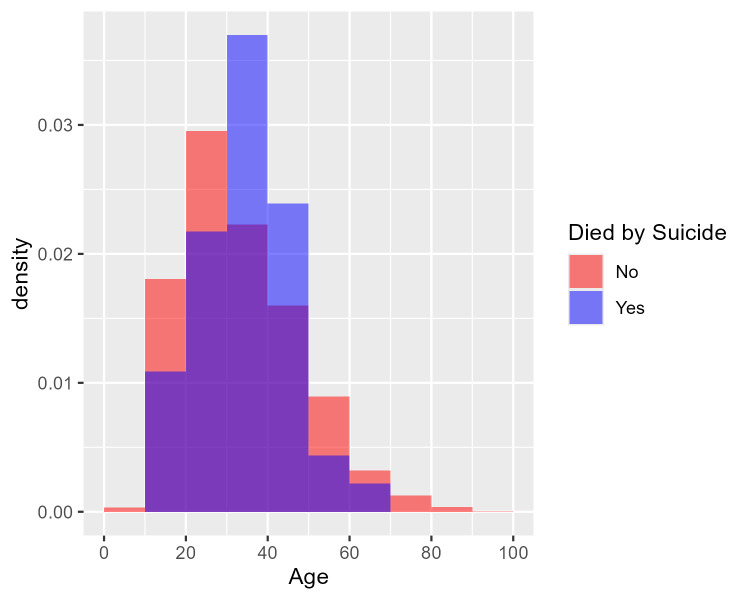
Age distribution of health care consumers who did and did not die by suicide. Figure 2 shows the age distribution of health care consumers who did and did not die by suicide using two overlaid histograms. From the logistic regression findings, purple area in each plot denotes overlap between the two overlaid histograms. Unlike Figure 1, the blue histogram’s density is concentrated in the middle, suggesting that health care consumers who died by suicide were particularly concentrated in the 30-50 age group. Age was not a predictor of occurrence for ‘Death by Suicide’ ( *p = 0.3089*), however using a quadratic age term to check for non-monotonic relationships it was found in the ‘Suicide’ model, both the linear ( *p =* 0.00388) and quadratic ( *p =* 0.00681) terms were statistically significant. This suggests that the risk of death by suicide among healthcare consumers increase with age until a peak age of 41 and decreases thereafter. From 3 years (2017-2019) of retrospective data, the median age for suicide was 36 years old.

This suggests that the risk of death by suicide among health care consumers increase with age until a peak age of 41 and decreases thereafter. The median ages for death by suicide and attempted suicide were 36 years and 30 years, respectively. Figure 1 shows the age distribution of health care consumers who had and had not attempted suicide using two overlaid histograms. Figure 2 similarly shows the age distribution of health care consumers who had and had not died by suicide. The purple area in each plot denotes overlap between the two overlaid histograms. The two figures cohere with the logistic regression findings: there is no visible difference between the red and blue histograms in Figure 1; hence no evidence of a difference in age distribution between those who attempted suicide and those who did not. In Figure 2, however, the blue histogram’s density is concentrated in the middle, suggesting that health care consumers who died by suicide were particularly concentrated in the 30-50 age group.

Using the Pearson Chi-square test of independence, it was found that the *p*-value was *< 0.05* for the associations between gender and attempted suicide ( *p = 0.004484*) and gender and death by suicide ( *p = 1.716 × 10 ^−8^*), suggesting males are more likely than females to die by suicide and attempt suicide. A logistic regression model was also fitted, with gender as the independent variable. Gender was again found to be a statistically significant predictor of both attempted suicide ( *p* = 0.00362) and death by suicide ( *p =* 1.78 × 10 ^−7^). The logistic regression model also allowed for computation of expected odds ratios. The odds of males attempting suicide were found to be 2.053 times as high as the odds of females attempting suicide, while the odds of males dying by suicide were found to be 5.049 times as high as those of females ( Tilley et al., 2023).

To analyse possible relationships between gender and age and the method of (attempted) suicide, cases of attempted suicide and death by suicide were combined to increase the frequencies. There were then 133 cases of asphyxiation/hanging, 22 cases of overdose or poisoning, and 28 cases of other or unspecified methods. Due to this response variable having three categories, a multinomial logistic regression model ( Table 3) was fitted with method of death by suicide/attempted suicide as a response variable and age and gender as independent variables, with an interaction of age and gender as well. No statistically significant coefficient predictors were found in the model at the 5% level. Looking at the method of death by suicide/attempted suicide vs. gender using Fisher’s Exact Test ( Table 4), there was a statistically significant relationship ( *p = 0.0005098*), specifically, it appears that males are more likely to use asphyxiation/hanging, while females are more likely to use poisoning or overdose.

**Table 3.** Multinomial logistic regression to predict Death by Suicide or Attempted Suicide method by age and gender.

*Coefficients:*				
**Method**	**(Intercept)**	**Age**	**Male**	Age * Male
*Other or unspecified*	-1.278676	0.02476155	0.9989150	0.01170249
*Asphyxiation/hanging*	1.704831	-0.03863174	0.5557824	0.04890228
*Std. Errors:*				
**Method**	**(Intercept)**	**Age**	**Male**	Age * Male
*Other or unspecified*	1.2664027	0.02880904	2.380916	0.06678731
*Asphyxiation/hanging*	0.9889576	0.02636292	2.006309	0.05978044
*p-values *				
**Method**	**(Intercept)**	**Age**	**Male**	Age * Male
*Other or unspecified*	0.3126430	0.3900612	0.6748137	0.8609066
*Asphyxiation/hanging*	0.0847319	0.1428169	0.7817668	0.4133392

Table 3 presents the results from a multinominal logistic regression model used to predict death by suicide or attempted suicide method by age and gender. To analyse possible relationships between gender and age and the method of (attempted) suicide, cases of attempted suicide and death by suicide were combined to increase the frequencies. The multinomial logistic regression model was fitted with method of suicide/attempted suicide as a response variable and age and gender as independent variables, with an interaction of age and gender as well. No statistically significant coefficient predictors were found in the model at the 5% level. There were 133 cases of strangulation, 22 cases of overdose or poisoning and 28 cases of other or unspecified method.

**Table 4.** Fisher’s Exact Test – Gender association to type of Death by Suicide.

Gender	Overdose/poisoning	Other/Unspecified	Asphyxiation/hanging
Female	59.1%	35.7%	15.8%
Male	22.7%	35.7%	48.1%
Unknown	18.2%	28.6%	36.1%

p-value = 0.0005098; Alternative hypothesis: two.sided.

Table 4 presents the results of Fisher’s Exact Test, used to test for an association between gender and type of death by suicide, for the 3-year (2017-2019) census data. Looking at the method of death by suicide/attempted suicide vs. gender using Fisher's Exact Test, there was a statistically significant relationship ( *p = 0.0005098*), specifically, it appears that males are more likely to use strangulation, while females are more likely to use poisoning or overdose from the study site.

An ANOVA was also run to check for differences in mean age of victims (dependent variable) across the three methods of death by suicide or attempted suicide ( Table 5). Statistically significant differences between groups were identified ( *p = 0.0333*). Using Tukey’s HSD method for post hoc comparisons ( Table 6), it was found that the mean age of asphyxiation/hanging victims is less than the mean age of “Other or unspecified” victims ( *p = 0.0414684*) ( Tilley, 2025).

**Table 5.** ANOVA – Age relationship with method of Attempted Suicide or Death by Suicide.

Term	Degree of Freedom	Sum of Squared Residuals	Mean Squared	F-value	p-value
Method of Death by Suicide	2	1017.113	508.5565	3.509373	0.0333395
Residuals	109	15795.601	144.9138		

Table 5 presents results for an Analysis of Variance (ANOVA) that was run to check for differences in mean age of victims (dependent variable) across the three methods of death by suicide or attempted suicide. Statistically significant differences between groups were identified at *p = 0.0333* from the census data over the 3-year period (2017-2019).

**Table 6.** Tukey’s HSD Results - Differences in Mean Age by Method of Attempted Suicide or Death by Suicide.

Comparison	Difference in Means	Lower Confidence Limit	Upper Confidence Limit	Adjusted p-value
Other or unspecified-overdose or poisoning	2.750000	-6.543301	12.0433011	0.76215151
Asphyxiation/hanging-overdose or poisoning	-4.689189	-12.206652	2.8282732	0.30343063
Asphyxiation/hanging-other or unspecified	-7.439189	-14.647982	-0.2303966	0.04146837

Table 6 presents the results for Tukey’s Honest Significant Difference (HSD) test. This method is based on a studentized rage statistic and is used in connection with analysis of variance (ANOVA) as a post hoc method to identify pairwise significant differences. Using Tukey’s HSD method for post hoc comparisons, it was found that the mean age of strangulation victims is less than the mean age of “Other or unspecified” victims ( *p = 0.0414684*) from the census data over the 3-year period (2017-2019).

## Discussion

There was a range of death by suicide and suicidality typologies in the Garden Route District over the 3-year study period, presenting in 14% ( *n* = 412) of IMRs sampled (representing health care consumer engagement). Death by suicide took place on average 2.8 ( *n *= 102) times a month, and attempted suicide 2.3 ( *n *= 83) times a month, and roughly 6.3 ( *n* = 227) health care consumers presented to the WCEMS with suicidal ideation monthly. Notably 63% ( *n* = 1890) of IMRs (health care consumers) presented with mental illness sequela, often related to suicide and suicidality victims’ medical history ( Klonsky et al., 2016; Kułak-Bejda et al., 2021). Significantly, in the same sample, males were five and two times more likely to die by suicide and attempt suicide than females, respectively ( Tilley et al., 2023). Males appeared more likely to use asphyxiation/hanging, while females used overdose or poisoning as a means of death by suicide in the study site ( Tilley et al., 2023). This dataset identified the risk of death by suicide, in a LMIC, to increase with age, until a peak age of 41 and decreases thereafter, making death by suicide risk the highest at 41 years of age. The median ages for death by suicide and attempted suicide were 36 years and 30 years, respectively. Notably in LMICs, suicide is a major public health issue, with 77% to 88% of global suicides occurring in these settings. While suicide rates increase with age in many regions, LMICs show a significant, distinct peak in suicide mortality among young adults, particularly those aged 15–29 years and 30–49 years ( Vijayakumar et al., 2016).

The data analysis provided similar inferences already denoted in articles on suicide globally, with asphyxiation/hanging being the most common method, and men being the most likely to die by suicide ( Klonsky et al., 2016; Kootbodien et al., 2020; Rahman et al., 2017; Ritchie et al., 2015). However, the presence of suicidal ideation, attempted suicides, cutting self-harm and overdose/DSP in the dataset is what was illuminating. Understandably, antecedents for suicidality ( Klonsky et al., 2016; Lim et al., 2019), individuals who inflict non-suicidal self-injury (disorder) are at risk of suicide attempts ( Brager-Larsen et al., 2024), while suicidal ideation and progression into suicide attempts are two phenomena that produce predictors towards death by suicide ( World Health Organization, 2014). Using the ideation-to-action framework, literature suggests deliberate self-harm and depression to be early and accurate indicators for suicidal ideation and suicidality ( Klonsky et al., 2016). The mental illness sequela from the dataset could suggest that, through better medical surveillance, more effort could be put into early suicide detection, knowing that PTSD, bipolar disorder, depression, substance abuse and suicidal ideation are associated with suicide deaths ( Klonsky et al., 2016). Prehospital emergency medical care provides health action to health conditions through emergency medicine in a time sensitive approach with universality and responsivity ( Christopher et al., 2014; Naidoo, 2017; Tilley et al., 2023) and can recognise the patterns and needs of mental healthcare consumers from an early stage to interrupt suicidality and limit access to methods of harm ( Florentine & Crane, 2010).

This dataset does not explain the perceptions prehospital EC providers have towards health care consumers who have suicidal ideation, suicidality and have attempted suicide. In various articles prehospital EC providers have explained to feel misconstrued on the concept of mental illness, self-harm and suicidality, often feeling lost and depleted by lack of legislation and policy, treatment protocols, training, guidance and personal negative conflict on own perceptions of attending mental health emergencies rather than trauma/medical emergencies ( Evans et al., 2018; O’Sullivan, 2014; Rees et al., 2015, 2018; Stander et al., 2021). In a study done in the same province in SA, it was found that 80% of the prehospital EC providers in the study had no prior training to manage suicidal health care consumers, seldom using formal suicide evaluation and capacity check tools, while implying negative feelings and connotations towards attempted suicide victims ( Evans et al., 2018). Inevitably, this lack of compassion, training and knowledge provides a precarious situation for prehospital EC providers, as this could prevent early mental health surveillance and suicidality interruption. A lack of praxis and management of suicidality and death by suicide caseload could put the EC provider helpless, creating an emotional backlash with direct or vicarious traumatisation to the EC provider. Notably, prehospital EC providers have disclosed battling with lasting visions from death by suicide scenes and battling with anxiety, PTSD and depression ( Padmanabhanunni & Pretorius, 2025; Rothes et al., 2020).

WCEMS prehospital EC providers locate in the suicide and suicidality burden and need to consider analytical clinical decision making ( Emond et al., 2024) in managing and treating mental healthcare consumers while considering the societal, cultural, religious and socioeconomic risk factors synonymous with suicide in SA ( Kootbodien et al., 2020). SA is precariously placed in the mental health milieu, with deinstitutionalisation and no appropriate policy and compensatory community mental health care services created ( Motsoaledi & Matsoso, 2013; World Health Organization, 2003). Atrocities from apartheid, poverty and inequality create exponential societal risk levels for suicidality and suicide, placing the prehospital EC provider and EMS at the forefront of mental health and suicidality emergencies. Located in the forefront of this caseload, prehospital EC providers need to have capacity to manage, treat and transport these health care consumers, have potential to interrupt suicidality by limiting access to harmful methods ( Florentine & Crane, 2010), contribute to social capital through latent capacity ( Tilley et al., 2023) while minding risk of direct and vicarious self-traumatization ( Sandford et al., 2021).

This socioeconomic landscape renders the concept of further research into Trauma Informed Care (TIC) as an interlude to disrupt negative postulation to suicidality, whereby understanding that childhood traumatic experiences can show signs of future mental health challenges, as most (mental) health is affected by past trauma ( Melillo et al., 2025; SAMHSA, 2014). Understanding and researching the benefits of TIC could be purposeful in an approach to management and training for healthcare consumers and EC providers respectively. The illumination of TIC and a syndemic approach to suicide could be associated with a dignified response to managing a marginalized group and the risk of vicarious traumatization of the EC provider ( Sandford et al., 2021). Subsequently, a syndemic approach to suicide ( Caulkins, 2022) in Southern Africa could be associated with interrupting suicidality, protecting people living (and dying) with such risk. Suicidality and suicide phenomenon is a complexity of challenges and the syndemic approach shows intersectionality to these challenges. The syndemic approach looks at suicide as a combination of challenges like culture, poverty, socioeconomic circumstances, mental illness, violence, trauma, chronic pain and stigma which amplify each other ( Caulkins, 2022). By manifesting research into the syndemic approach, the EMS could reflect this approach through training EC providers to identify suicidality as a product of behavioral, clinical and social challenges whereby TIC, medical treatment and referral pathways are tailored to mitigate risk of harm.

## Conclusion

Tilley et al. (2023) described the Deliberate Self-Harm burden for the prehospital EMS; however, the novelty in this paper lies in the typological disaggregation of suicidality and death by suicide within EMS records. The authenticity of this research elucidates the suicidality burden faced by the South African prehospital EMS. Death by suicide and suicidality typology in the EMS have not been previously assessed in SA, illuminating a research and practice problem space. This study describes the prehospital suicide and suicidality burden for the WCEMS. Prehospital EC providers need to retain the praxis, training, emotion, policies and legislation to comprehensively manage, treat and transport healthcare consumers with suicidality ( Simpson et al., 2025) and that this proven challenge could require lateral deliberation ( Emond et al., 2024). This documented death by suicide and suicidality typology presents a novel and fundamental understanding of the prehospital suicidality problem space definition. This study quantifies the burden for the EMS; however, it provides no solution to training, management, treatment, or EC provider perception towards death by suicide and suicidality. Further study is required on EC provider stigmatisation towards death by suicide and suicidality, while auditing the need to assess policy, praxis, medical surveillance, EC provider clinical competency capacity and suicidality victim perspectives, as healthcare consumer level interventions on strategic suicide prevention have aided in the reduction of suicide attempts ( Zarska et al., 2023).

### Limitations of the study

Emergency call takers are not trained mental health care professionals and do not make mental health diagnoses, while the vernacular of the healthcare consumers provide a challenge in reporting. The inherent risk of retrospective medical records includes misclassification bias, e.g., where a case was incorrectly categorised as death by suicide when it was death by some other cause, or vice versa. Retrospective data limitations also apply. Risk of type one error is mitigated by the significance level and large sample size. This dataset does not elucidate perceptions of prehospital EC providers towards suicidality and relies on call taker and dispatcher reporting. Notably there is a lack of post event outcome verification, such as post-mortem data.

## Ethical considerations

Ethics was granted for this study through the Research Ethics Committee of the Cape Peninsula University of Technology *(CPUT/HW-REC 2019/H17).* Permission for access to the Western Cape National Health Research Database was obtained from the Western Cape Government *(WC_201911_033).*

## Data Availability

Figshare: Access to health care for health care consumers with mental health needs: an Emergency Medical Service perspective. https://doi.org/10.6084/m9.figshare.30392569. The project contains raw retrospective data in an Excel spreadsheet with analysed data in graphs and tables using R statistical software, Binary and multinomial logistic regression, Pearson’s Chi-squared test of independence, Fisher’s Exact Test, Analysis of Variance (ANOVA), and Tukey’s Honest Significant Difference (HSD). A dissertation with full analysis is also provided. Data are available under the terms of the Creative Commons Attribution 4.0 International licence (CC-BY 4.0) ( Tilley, 2025).
