# Supplementary material for: Typologies of suicidality and suicide presenting to a prehospital South African Emergency Medical Service: a retrospective cross-sectional analysis
Source: F1000Res. 2026 Mar 4;14:1201. Originally published 2025 Nov 3. [Version 3] doi: 10.12688/f1000research.171712.3 (PMC12921398; doi:10.12688/f1000research.171712.3)
Supplement: Peer review history

# Reviewer report: Reviewer response for version 3

**DOI:** 10.5256/f1000research.197040.r464405

**Published:** 2026-03-06

**Authors:** Wesley Craig

**Affiliations:** 1 University of Cape Town Division of Emergency Medicine (Ringgold ID: 536985), Cape Town, Western Cape, South Africa

Well done to the authors on a well presented manuscript. I have no further comments.

Is the work clearly and accurately presented and does it cite the current literature?

Yes

If applicable, is the statistical analysis and its interpretation appropriate?

Yes

Are all the source data underlying the results available to ensure full reproducibility?

Yes

Is the study design appropriate and is the work technically sound?

Yes

Are the conclusions drawn adequately supported by the results?

Partly

Are sufficient details of methods and analysis provided to allow replication by others?

Yes

Reviewer Expertise:

NA

I confirm that I have read this submission and believe that I have an appropriate level of expertise to confirm that it is of an acceptable scientific standard.

# Reviewer report: Reviewer response for version 3

**DOI:** 10.5256/f1000research.197040.r464406

**Published:** 2026-03-05

**Authors:** Richard Mottershead

**Affiliations:** 1 College of Nursing, University of Baghdad, Baghdad, Iraq

Dear Authors,

 Thank you for your careful and considered responses to the points raised during the review process. I appreciate the clarity and diligence with which you have addressed each comment and strengthened the manuscript accordingly.

 The revised paper, *Typologies of suicidality and suicide presenting to a prehospital South African Emergency Medical Service: a retrospective cross-sectional analysis*, makes a notable contribution to the field. By examining patterns of suicidality within the prehospital EMS context, the study adds valuable knowledge to an under-researched area and increases awareness of the complexity and burden of suicide-related presentations in emergency care settings.

 The work has clear relevance for clinical practice, service planning, and future research, particularly within resource-constrained and high-demand healthcare environments. It provides meaningful insights that can inform training, policy development, and targeted prevention strategies.

 Thank you for your thoughtful revisions and for contributing important evidence to this critical area of public health and emergency care.

 Kindest regards,

 Dr. Richard Mottershead

Is the work clearly and accurately presented and does it cite the current literature?

Partly

If applicable, is the statistical analysis and its interpretation appropriate?

Yes

Are all the source data underlying the results available to ensure full reproducibility?

Yes

Is the study design appropriate and is the work technically sound?

Partly

Are the conclusions drawn adequately supported by the results?

Partly

Are sufficient details of methods and analysis provided to allow replication by others?

Partly

Reviewer Expertise:

My area of research is in mental health, societial challenges, applied research

I confirm that I have read this submission and believe that I have an appropriate level of expertise to confirm that it is of an acceptable scientific standard.

# Reviewer report: Reviewer response for version 2

**DOI:** 10.5256/f1000research.194504.r458179

**Published:** 2026-02-19

**Authors:** Wesley Craig

**Affiliations:** 1 University of Cape Town Division of Emergency Medicine (Ringgold ID: 536985), Cape Town, Western Cape, South Africa

Thanks so much for the opportunity to review. The paper certainly addresses an important and under-researched area in South African out-of-hospital emergency care and its intersection with mental health care provision.

 This study examined the burden and typology of suicidality encountered by EMS in the Garden Route District of the Western Cape, South Africa. It used a retrospective cross-sectional analysis of EMS records from 2017-2019. From 413,712 EMS records, the authors identified 2,976 mental-health-related incidents, of which 412 involved suicidality, including suicidal ideation, attempted suicide, and death by suicide. The study found gender differences in suicide method and outcomes. Men were significantly more likely to die by suicide than women, and strangulation was the most common method among men, while poisoning or medication overdose was more common among women. The authors conclude that suicidality represents a meaningful but under-recognised component of the EMS caseload in South Africa. Improved training, policy guidance, mental-health surveillance, and research into EMS provider experiences and stigma was recommended. I believe the study is conceptually strong and publishable with minor-to-moderate revision.

- The research aim could be stated explicitly, aside from the abstract, it seems to be narratively embedded in the background. This also goes for the objectives; they don’t seem to be listed clearly anywhere.
- It would be helpful to the readership to state earlier that the case descriptions are based on EMS call-taker and call-categories not postmortem findings. This is especially important when determining intent in terms of “Death by suicide” vs accidental death and homicide. This is brought up in limitations, but should come earlier in methods and briefly in discussion (in general the misclassification bias/call-taker judgment etc).
- While the analysis and results appear robust, the level of statistical explanation is more detailed than necessary for most journals and at times reads like a dissertation. This can be quickly addressed by summarising the rationale for each test then focussing on interpretation rather than statistical pedagogy.
- Please refrain from citing papers which are not directly relevant to the statements (e.g. “The null hypothesis (H0) states ‘there is no association between two variables while the alternative hypothesis (Ha) states, there is an association between two variables (Bolboacă et al., 2011, p. 530; Tilley, 2021; Tilley et al., 2023).” The latter two references are not papers informing methods/analytic approaches. There are quite a few of these in the manuscript, please look out for these throughout.
- I would suggest that references to previous publications using the same dataset should be moved from the Results section to the Introduction or Discussion, and the novelty of the present analysis should be stated more clearly.
- A significant amount of time is spent on “age” (41 years being peak risk) as a variable yet there is no mention of it in the discussion. It would be useful to link this to other literature as it remains without context here.
- The discussion is strong and statements suggesting that EMS adopt TIC approaches is almost certainly valid, however it does move beyond the descriptive epidemiological nature of the manuscript. These can rather be framed as implications for users, or just areas for future research.
- Syndemic is a novel point of discussion and has value here however it seems awkwardly placed in the introduction, with nothing in the discussion, then brought back into the conclusion. “…however, the novelty in this paper provides and removes the DSH scope and focuses on prehospital suicidality typology while suggesting a syndemic approach to suicide in the African setting.“ I don’t believe it does this in its current form. Some more practical application could be valuable here, what would it look like if EMS training reflected this approach? The term is also inconsistently written in quotation marks.
- Trauma-informed care is also brought in at the latter end of the discussion with more explanation provided in the conclusion, this should be moved to the discussion section as no new information should be presented in the conclusion.
- Consent: The manuscript mentions “Western Cape Government National Department of Health Ethics Council (WC_201911_033)” I don’t believe this is the name of a single formal ethics committee. It appears to be a mislabelled or conflated reference to two different structures. That said, the ref number looks like a National Health Research Database (NHRD) number for a Western Cape Department of Health and Wellness Research site.
- More minor things
  - Western Cape Department of Health and Wellness – I believe they’ve changed from ‘Government’ to ‘Department’.
  - “healthcare” vs “health care”
  - Inconsistent capitalisation of “Death by suicide”
  - Limitations section: “don’t” – avoid the use of contractions, try to write in full.
  - There are quite a few grammar and spacing gremlins throughout, another proofread will be adequate to pick up on these. In particular the use of inappropriate capitalisation and the inconsistent use of abbreviations.

Is the work clearly and accurately presented and does it cite the current literature?

Yes

If applicable, is the statistical analysis and its interpretation appropriate?

Yes

Are all the source data underlying the results available to ensure full reproducibility?

Yes

Is the study design appropriate and is the work technically sound?

Yes

Are the conclusions drawn adequately supported by the results?

Partly

Are sufficient details of methods and analysis provided to allow replication by others?

Yes

Reviewer Expertise:

NA

I confirm that I have read this submission and believe that I have an appropriate level of expertise to confirm that it is of an acceptable scientific standard, however I have significant reservations, as outlined above.

# Response

**Published:** 2026-02-22

**Authors:** Daniel Tilley

**Affiliations:** Emergency Medical Science, Cape Peninsula University of Technology - Bellville Campus, Bellville, Western Cape, South Africa

Please see comments in Italics. We would like to thank the reviewer for the suggestions and advice in making this article more academically sound for international audiences and the journal as a whole. Thank you

- The research aim could be stated explicitly, aside from the abstract, it seems to be narratively embedded in the background. This also goes for the objectives; they don’t seem to be listed clearly anywhere.

*This has been done accordingly*

- It would be helpful to the readership to state earlier that the case descriptions are based on EMS call-taker and call-categories not postmortem findings. This is especially important when determining intent in terms of “Death by suicide” vs accidental death and homicide. This is brought up in limitations, but should come earlier in methods and briefly in discussion (in general the misclassification bias/call-taker judgment etc).

*This has been done accordingly and added earlier in materials and methods*

- Please refrain from citing papers which are not directly relevant to the statements (e.g. “The null hypothesis (H0) states ‘there is no association between two variables while the alternative hypothesis (Ha) states, there is an association between two variables (Bolboacă et al., 2011, p. 530; Tilley, 2021; Tilley et al., 2023).” The latter two references are not papers informing methods/analytic approaches. There are quite a few of these in the manuscript, please look out for these throughout.

*this has been fixed and changed*

- I would suggest that references to previous publications using the same dataset should be moved from the Results section to the Introduction or Discussion, and the novelty of the present analysis should be stated more clearly.

*This has been done in the discussion*

- A significant amount of time is spent on “age” (41 years being peak risk) as a variable yet there is no mention of it in the discussion. It would be useful to link this to other literature as it remains without context here.

*This has been added and discussed in discussion, thank you.*

- The discussion is strong and statements suggesting that EMS adopt TIC approaches is almost certainly valid, however it does move beyond the descriptive epidemiological nature of the manuscript. These can rather be framed as implications for users, or just areas for future research.

*This has been done accordingly to this suggestion*

- Syndemic is a novel point of discussion and has value here however it seems awkwardly placed in the introduction, with nothing in the discussion, then brought back into the conclusion. “…however, the novelty in this paper provides and removes the DSH scope and focuses on prehospital suicidality typology while suggesting a syndemic approach to suicide in the African setting.“ I don’t believe it does this in its current form. Some more practical application could be valuable here, what would it look like if EMS training reflected this approach? The term is also inconsistently written in quotation marks.

*This has been defined better and fixed accordingly*

- Trauma-informed care is also brought in at the latter end of the discussion with more explanation provided in the conclusion, this should be moved to the discussion section as no new information should be presented in the conclusion.

*This has been changed accordingly and removed from the conclusion*

- Consent: The manuscript mentions “Western Cape Government National Department of Health Ethics Council (WC_201911_033)” I don’t believe this is the name of a single formal ethics committee. It appears to be a mislabelled or conflated reference to two different structures. That said, the ref number looks like a National Health Research Database (NHRD) number for a Western Cape Department of Health and Wellness Research site.

*This has been changed as advised. Thank you for this advice*

- More minor things
  - Western Cape Department of Health and Wellness – I believe they’ve changed from ‘Government’ to ‘Department’.
  - “healthcare” vs “health care”
  - Inconsistent capitalisation of “Death by suicide”
  - Limitations section: “don’t” – avoid the use of contractions, try to write in full.
  - There are quite a few grammar and spacing gremlins throughout, another proofread will be adequate to pick up on these. In particular the use of inappropriate capitalisation and the inconsistent use of abbreviations.

*Thank you for this advice. this has all been attended to and corrected as suggested.*

# Reviewer report: Reviewer response for version 2

**DOI:** 10.5256/f1000research.194504.r457459

**Published:** 2026-02-17

**Authors:** Richard Mottershead

**Affiliations:** 1 College of Nursing, University of Baghdad, Baghdad, Iraq

I would like to thank the authors for this opportunity to peer review their article.  

**Summary**

 This study presents a retrospective cross-sectional analysis of suicidality and suicide cases recorded in the Western Cape Government Emergency Medical Services (WCGEMS) Incident Management Records over a three-year period (2017–2019) in the Garden Route District of South Africa.

 From 413,712 total EMS records, 2,976 mental health-related cases were identified, of which 412 (14%) involved suicidal ideation, attempted suicide, or death by suicide. The study provides typological analysis of suicide methods, gender associations, and age distributions using logistic regression, multinomial regression, ANOVA, and Fisher’s Exact Test. The findings indicate 

 - An average of 2.8 deaths by suicide and 2.3 attempted suicides per month.

 - Males were approximately 5 times more likely to die by suicide than females.

 - Strangulation was the most common method among males; poisoning/overdose predominated among females.

 - Risk of death by suicide appeared to peak at approximately age 41 (quadratic model).

 The study aims to quantify and typologise suicidality within the prehospital EMS context and suggests a syndemic framework for understanding suicide within South Africa’s socio-economic landscape.

 The topic is highly relevant, particularly in low- and middle-income country contexts where prehospital suicide research is limited. The dataset is substantial, and the statistical analyses are appropriately selected for the research aims.

**Overall Assessment**

 This manuscript makes an important contribution by quantifying the prehospital suicidality burden within a rural South African EMS context. The dataset is large, the statistical methods are generally appropriate, and the findings are clearly reported.

 However, my suggestions would be that the authors review these several areas which would (I believe) benefit from clarification and strengthening before full approval. These relate primarily to:

- Conceptual framing
- Operational definitions
- Interpretation of statistical findings
- Overextension of conclusions
- Clarity around “novelty” claims

These are refinements rather than major corrections but would enhance the hard work and efforts of the research team. 

**Recommendations**

**1. Clarify Operational Definitions and Case Classification**

 The manuscript relies on EMS categorisation (e.g., “Self-Harm-other,” “Self-Harm-poisoning,” “Psychiatric/Behavioural Problems”), which are not clinical diagnoses.

 To strengthen methodological clarity, the authors should:

 Provide clearer operational definitions of:

- Suicidal ideation
- Attempted suicide
- Death by suicide
- Explain how misclassification bias may influence estimates.
- Clarify how “parasuicidal” behaviour was differentiated from attempted suicide.

I might suggest that call-takers are not mental health professionals, this is a critical limitation and should be foregrounded more clearly in the methods and limitations sections. Perhaps the call-takers had enhanced training which was not highlighted within the article? 

**2. Strengthen Justification of the “Syndemic” Framing**

 The syndemic concept is introduced as a major theoretical contribution; however, it is not analytically tested within the dataset.

 To strengthen conceptual coherence, the authors should:

- Clarify whether “syndemic” is used as:
- A theoretical lens,
- A hypothesis-generating concept,
- Or a demonstrated empirical finding.
- Avoid implying that syndemic relationships were statistically examined unless explicitly tested.
- Consider moderating the claim of novelty unless the framework is analytically integrated into results.

Currently, the syndemic argument is persuasive but somewhat aspirational rather than empirically grounded in the presented analysis.

**3. Moderate Claims of Novelty**

 The manuscript states that suicide typology in the EMS has “not been previously assessed in South Africa.”

 This should be carefully contextualised:

- I note that there is prior work (including by the authors) on deliberate self-harm in EMS.
- The novelty lies in the typological disaggregation of suicidality and death by suicide within EMS records.

Refining the novelty claim would enhance academic credibility and whilst also highlighting the authors previous contributions.

**Conclusion**

 This study provides valuable epidemiological insight into suicidality and suicide presenting to a rural South African EMS. The methodology is broadly appropriate, the findings are coherent, and the topic is highly relevant.

 I believe that if these recommendations are followed the manuscript will be suitable for full approval. I will be available for a quick turn around. 

 Thank you

 Dr. Richard Mottershead

Is the work clearly and accurately presented and does it cite the current literature?

Partly

If applicable, is the statistical analysis and its interpretation appropriate?

Yes

Are all the source data underlying the results available to ensure full reproducibility?

Yes

Is the study design appropriate and is the work technically sound?

Partly

Are the conclusions drawn adequately supported by the results?

Partly

Are sufficient details of methods and analysis provided to allow replication by others?

Partly

Reviewer Expertise:

My area of research is in mental health, societial challenges, applied research

I confirm that I have read this submission and believe that I have an appropriate level of expertise to confirm that it is of an acceptable scientific standard, however I have significant reservations, as outlined above.

# Response

**Published:** 2026-02-22

**Authors:** Daniel Tilley

**Affiliations:** Emergency Medical Science, Cape Peninsula University of Technology - Bellville Campus, Bellville, Western Cape, South Africa

Please note responses are given in italics. We would like to thank the reviewer for the advice and suggestions in making this document more academically sound for international audiences and the journal. Thank you for your review.

**Recommendations**

**1. Clarify Operational Definitions and Case Classification**

 The manuscript relies on EMS categorisation (e.g., “Self-Harm-other,” “Self-Harm-poisoning,” “Psychiatric/Behavioural Problems”), which are not clinical diagnoses.

 To strengthen methodological clarity, the authors should:

 Provide clearer operational definitions of:

- Suicidal ideation
- Attempted suicide
- Death by suicide
- Explain how misclassification bias may influence estimates.
- Clarify how “parasuicidal” behaviour was differentiated from attempted suicide.

*Operational definition have been placed in accordingly in the appropriate places. Misclassification bias has been defined and explained in the methodology and in the limitations. Parasuicidal has been differentiated accordingly to explain this definition accurately.*

 I might suggest that call-takers are not mental health professionals, this is a critical limitation and should be foregrounded more clearly in the methods and limitations sections. Perhaps the call-takers had enhanced training which was not highlighted within the article? 

*This has been corrected and explained accordingly how dispatchers make us of caller prompts, senior medical technicians and senior paramedics to help with patient understandings. This is brought up earlier in the materials and methods sections.*

**2. Strengthen Justification of the “Syndemic” Framing**

 The syndemic concept is introduced as a major theoretical contribution; however, it is not analytically tested within the dataset.

 To strengthen conceptual coherence, the authors should:

- Clarify whether “syndemic” is used as:
- A theoretical lens,
- A hypothesis-generating concept,
- Or a demonstrated empirical finding.
- Avoid implying that syndemic relationships were statistically examined unless explicitly tested.
- Consider moderating the claim of novelty unless the framework is analytically integrated into results.

Currently, the syndemic argument is persuasive but somewhat aspirational rather than empirically grounded in the presented analysis.

*This has been described better in the text as a theoretical lens and better explained in the discussion and moves away from the suggestion that this concept was analyzed.*

**3. Moderate Claims of Novelty**

 The manuscript states that suicide typology in the EMS has “not been previously assessed in South Africa.”

 This should be carefully contextualised:

- I note that there is prior work (including by the authors) on deliberate self-harm in EMS.
- The novelty lies in the typological disaggregation of suicidality and death by suicide within EMS records.

Refining the novelty claim would enhance academic credibility and whilst also highlighting the authors previous contributions.

*Thank you so much for this advice. and for providing us with a better novelty claim. This has been amended accordingly*

# Reviewer report: Reviewer response for version 2

**DOI:** 10.5256/f1000research.194504.r446906

**Published:** 2026-01-23

**Authors:** Paul Sookram

**Affiliations:** 1 University of the Free State, Bloemfontein, Free State, South Africa

This manuscript addresses an important and under-researched EMS public health issue using a large retrospective dataset. The methodology is appropriate, and the analyses are robust. Minor revisions are required to improve language clarity, methodological transparency, and consistency in terminology and statistical reporting

 2. Language, spelling, and style (minor but frequent issues)

 General comments

 The manuscript is generally readable, but there are numerous typographical, spacing, and grammatical inconsistencies. Several errors appear repeatedly and should be corrected globally.

 Key issues and examples

 Spacing and typographical errors

 Examples:

 “Southj african” → South African

 “theAfrica region” → the Africa region

 “attemptedmurders” → attempted murders

 “healthcare” vs “health care” → choose one and apply consistently (journal style usually prefers health care).

 “multinomial” misspelled as multinominal in several places.

 Inconsistent capitalisation

 “Death by Suicide”, “Attempted Suicide”, “Suicide” are capitalised inconsistently.

 Recommendation: only capitalise when starting a sentence or when formally defining a variable.

 Long and complex sentences

 Some paragraphs (especially Introduction and Discussion) would benefit from sentence shortening to improve clarity.

 Example: paragraphs discussing social determinants and syndemic theory could be split.

 Redundancy

 Repetition of phrases such as “prehospital Emergency Medical Services” could be reduced after first definition (EMS).

 Recommendation:

 A professional language edit or journal copy-edit pass would substantially improve readability but is not a fatal flaw.

 3. Introduction and literature framing

 Strengths

 Strong contextualization of suicide as a public health problem in South Africa. Effective linkage between social determinants, mental health systems, and EMS burden.The introduction of syndemic theory is novel and relevant.

 Areas for improvement

 Overcrowding of statistics

 The Introduction contains many statistics in a short span. Suggest prioritising key figures and moving some contextual data to later paragraphs.

 Clearer research gap

 The research gap is implied but could be stated more explicitly: “There is limited empirical evidence describing suicidality typology within South African prehospital EMS settings.”

 Research question clarity

 The question is posed mid-paragraph.

 Recommend explicitly stating the aim and objectives at the end of the Introduction.

 4. Methodology

 Strengths

 Appropriate retrospective cross-sectional design.

 Census approach strengthens internal validity.

 Clear description of data source (IMRs). Ethical approvals are well documented. Methodological concerns and suggestions Inclusion/exclusion criteria

 Inclusion criteria rely heavily on dispatcher categorisation.

 This limitation is acknowledged later but should be more explicitly stated upfront.

 Definition of outcomes

 “Death by suicide” is defined as a prehospital service category, not forensic confirmation.

 This is appropriate but must be clearly emphasised earlier, especially for international readers.

 Use of multiple statistical tests

 While the analyses are appropriate, the rationale for each test could be more succinct. Consider a short summary table or paragraph explaining: Which tests answer which research questions.

 Multiple comparisons

 Given the number of tests run, there is risk of type I error. A brief statement acknowledging this limitation would strengthen the methodology.

 5. Results

 Strengths

 Results are comprehensive and logically ordered.

 Tables and figures support the narrative. Gender and method associations are clearly reported. Issues requiring correction or clarification Consistency in reporting percentages Percentages sometimes lack denominators. Always specify n/N for clarity.

 Terminology

 “Strangulation death” may be misinterpreted internationally.

 Suggest:

 “Strangulation (asphyxiation/hanging)” on first use.

 Age modelling

 The quadratic age analysis is well executed. However, the clinical or practical significance of the peak at age 41 could be briefly contextualised. Combining attempted suicide and suicide. Combining cases for multinomial analysis is justified statistically.This decision should be explicitly justified earlier in the Results section.

 6. Discussion

 Strengths

 Discussion aligns well with results.Good integration with international literature.

 Strong EMS-focused interpretation.The link to trauma-informed care is appropriate and timely.

 Areas for strengthening

 Balance

 Discussion occasionally leans toward advocacy. Consider tightening language to maintain a neutral scholarly tone.

 Causality- Avoid language that implies causation from cross-sectional data. Use phrases such as “associated with” rather than “leads to”.

 Syndemic framing- Strong conceptually, but could benefit from: A short paragraph explaining how EMS policy or training could operationalise a syndemic approach.

 7. Limitations

 Strengths-Limitations are acknowledged and appropriate.

 Suggested additions-Add explicit mention of: Potential misclassification bias. Reliance on dispatcher-reported categories. Lack of post-event outcome verification

 8. Referencing and citations

 Strengths

 Broad and relevant reference base. Good use of African and international literature.

 Issues- Inconsistency in formatting.Some references lack page numbers or consistent journal formatting.

 Grey literature-Media sources (e.g. Mail & Guardian, Action Society) are useful but should be clearly framed as contextual sources, not epidemiological evidence.

 Repetition-Some references appear multiple times in the reference list (likely formatting duplication

Is the work clearly and accurately presented and does it cite the current literature?

Yes

If applicable, is the statistical analysis and its interpretation appropriate?

Yes

Are all the source data underlying the results available to ensure full reproducibility?

Yes

Is the study design appropriate and is the work technically sound?

Yes

Are the conclusions drawn adequately supported by the results?

Yes

Are sufficient details of methods and analysis provided to allow replication by others?

Yes

Reviewer Expertise:

I am an expert in Health Professions Education

I confirm that I have read this submission and believe that I have an appropriate level of expertise to confirm that it is of an acceptable scientific standard.

# Response

**Published:** 2026-02-22

**Authors:** Daniel Tilley

**Affiliations:** Emergency Medical Science, Cape Peninsula University of Technology - Bellville Campus, Bellville, Western Cape, South Africa

Please see comments in Italics. We would like to thank the reviewer for all the positive suggestions and advice in making this article more academic sound and applicable to international standards for the journal. Thank you

 2. Language, spelling, and style (minor but frequent issues)

 General comments

 The manuscript is generally readable, but there are numerous typographical, spacing, and grammatical inconsistencies. Several errors appear repeatedly and should be corrected globally.

 Key issues and examples

 Spacing and typographical errors

 Examples:

 “Southj african” → South African

 “theAfrica region” → the Africa region

 “attemptedmurders” → attempted murders

 “healthcare” vs “health care” → choose one and apply consistently (journal style usually prefers health care).

 “multinomial” misspelled as multinominal in several places.

*Thank you for this report. This has been fix and changed accordingly.*

 Inconsistent capitalisation

 “Death by Suicide”, “Attempted Suicide”, “Suicide” are capitalised inconsistently.

 Recommendation: only capitalise when starting a sentence or when formally defining a variable.

*This has been changed in the document and corrected accordingly*

 Long and complex sentences

 Some paragraphs (especially Introduction and Discussion) would benefit from sentence shortening to improve clarity.

 Example: paragraphs discussing social determinants and syndemic theory could be split.

*The long paragraphs have been shortened in to smaller coherent sentences and split accordingly.*

 Redundancy

 Repetition of phrases such as “prehospital Emergency Medical Services” could be reduced after first definition (EMS).

*This has been corrected accordingly*

 Recommendation:

 A professional language edit or journal copy-edit pass would substantially improve readability but is not a fatal flaw.

 .

 Clearer research gap

 The research gap is implied but could be stated more explicitly: “There is limited empirical evidence describing suicidality typology within South African prehospital EMS settings.”

*This has been added. Thank you for pointing this out and aiding in a contextualizing sentence, that we added to the manuscript.*

 Research question clarity

 The question is posed mid-paragraph.

 Recommend explicitly stating the aim and objectives at the end of the Introduction.

*This has been rearranged and fixed accordingly.*

 4. Methodology

 This limitation is acknowledged later but should be more explicitly stated upfront.

 Definition of outcomes

 “Death by suicide” is defined as a prehospital service category, not forensic confirmation.

 This is appropriate but must be clearly emphasised earlier, especially for international readers.

*We added this in earlier as suggested*

 Use of multiple statistical tests

 While the analyses are appropriate, the rationale for each test could be more succinct. Consider a short summary table or paragraph explaining: Which tests answer which research questions.

 Multiple comparisons

 Given the number of tests run, there is risk of type I error. A brief statement acknowledging this limitation would strengthen the methodology.

*This is added and we explain how Tukey’s method is designed to control the familywise type I error rate at a fixed level (e.g., 0.05) despite the large number of pairwise comparisons being made.*

 Suggest:

 “Strangulation (asphyxiation/hanging)” on first use.

*This has been changed throughout document*

 Age modelling

 The quadratic age analysis is well executed. However, the clinical or practical significance of the peak at age 41 could be briefly contextualised. Combining attempted suicide and suicide. Combining cases for multinomial analysis is justified statistically.This decision should be explicitly justified earlier in the Results section.

*This is done accordingly*

 6. Discussion

 Areas for strengthening

 Balance

 Discussion occasionally leans toward advocacy. Consider tightening language to maintain a neutral scholarly tone.

 Causality- Avoid language that implies causation from cross-sectional data. Use phrases such as “associated with” rather than “leads to”.

*This has been done accordingly*

 Syndemic framing- Strong conceptually, but could benefit from: A short paragraph explaining how EMS policy or training could operationalise a syndemic approach.

*This has been added accordingly as suggested*

 7. Limitations

 Strengths-Limitations are acknowledged and appropriate.

 Suggested additions-Add explicit mention of: Potential misclassification bias. Reliance on dispatcher-reported categories. Lack of post-event outcome verification

*This has been done and explained in limitations and in methodology section. As suggested*

 8. Referencing and citations

 Issues- Inconsistency in formatting.Some references lack page numbers or consistent journal formatting.

*This is how the journal created the referencing format. APA was used as the initial reference format*

 Grey literature-Media sources (e.g. Mail & Guardian, Action Society) are useful but should be clearly framed as contextual sources, not epidemiological evidence.

*done accordingly*

 Repetition-Some references appear multiple times in the reference list (likely formatting duplication

*This duplication is in requirements of the journal for data availability and is part of the instructions to create a DOI and add a reference to the reference list.*
